# Jaranol: a compound with therapeutic activity against pathological cardiac remodelling via multi-target inhibition of the Snai3/TLR2 and NF-κB signaling pathways

**DOI:** 10.3389/fphar.2025.1723889

**Published:** 2026-01-02

**Authors:** Yao Zhang, Nan Li, Shiqi Chu, Heqing Fu, Xiaowen Yang, Huimin Xia, Yunfeng Xiao, Zhibin Xiao, Jing Liu, Yu Dong, Tianlong Liu

**Affiliations:** 1 Department of Pharmacy, Affiliated Hospital of Inner Mongolia Medical University, Hohhot, China; 2 Key Laboratory of Clinical and Basic Research on Cardiovascular Diseases, Basic Research Team of Cardiovascular Diseases, Affiliated Hospital of Inner Mongolia Medical University, Hohhot, China; 3 Department of Natural Medicinal Chemistry, College of Pharmacy, Inner Mongolia Medical University, Hohhot, China; 4 Center for New Drug Safety Evaluation and Research, Inner Mongolia Medical University, Hohhot, China; 5 Department of Clinical Pharmacy, College of Pharmacy, Inner Mongolia Medical University, Hohhot, China; 6 Engineering Technology Research Center of Pharmacodynamic Substance and Quality Control of Mongolian Medicine in Inner Mongolia, Hohhot, China

**Keywords:** jaranol, cardiac remodelling, Snai3/TLR2 signaling pathways, NF-κB signaling pathways, protective effect

## Abstract

Inhibiting pathological cardiac remodelling is considered an important therapeutic approach for heart failure, although effective strategies are still lacking in the clinic. Jaranol was found to be widely distributed in Chinese herbal medicine with multi biological functions, however, its effects on pathological cardiac remodelling remain unexplored. The present study identified the potential therapeutic effects of jaranol on cardiac remodelling base-joint analysis of the serum proteomic profile of patients with heart failure and *Astragalus membranaceus*-related potential targets. Jaranol treatment ameliorated angiotensin II (Ang II)- and transverse aortic constriction (TAC)-induced pathological cardiac remodelling *in vitro* and *in vivo,* as evidenced by the improved cardiac function, hypertrophy and fibrosis. The mRNA-sequencing and biochemical analyses showed that Toll-like receptor (TLR) signaling and TLR2 expression were suppressed in myocardial tissue after jaranol treatment. Mechanistically, jaranol enhanced the expression of transcription factor Snai3, leading to decreased expression of TLR2 in myocardium, meanwhile, adding Snai3 protein to culture media could suppressed TLR2 expression in neonatal mouse primary cardiomyocytes. Proteome microarray assays indicated that jaranol could interact with NF-κB, a key regulatory factor of the TLR signalling pathway. Indeed, jaranol suppressed the Ang II-induced translocation of NF-κB from the cytoplasm to the nucleus in human induced pluripotent stem (hiPS)-cardiomyocytes. In conclusion, the present study demonstrated a novel role of jaranol in preventing pathological cardiac remodelling via multi-target inhibition of Snai3/TLR2 and NF-κB signaling pathways.

## Graphical Abstract

**Figure:**
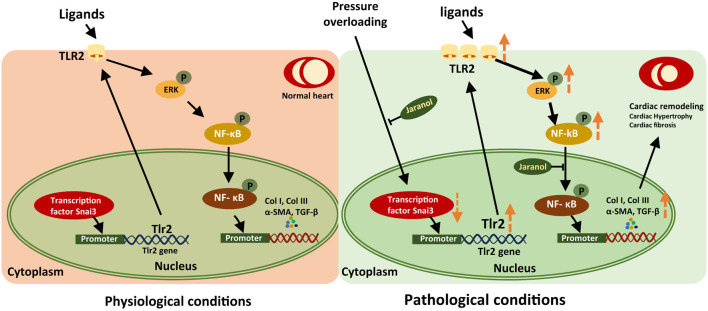
Jaranol ameliorates adverse cardiac remodelling via multi-target inhibition of Snai3/TLR2 and NF-κB signaling pathways.

## Introduction

Heart failure, a type of mortality equivalent to cancer, is the end stage of many cardiovascular diseases and a threat to life and health (Zhuang et al., 2022). Cardiac remodelling is defined as a compensatory and decompensated alteration of cardiac structure and function that occurs in response to chronic stress (aortic atherosclerosis or hypertension)/increased volume loading (aortic or mitral regurgitation), ischaemic injury (infarction), inflammatory diseases (myocarditis), and genetic mutations (Schnelle et al., 2021). The clinical presentation of pathological cardiac remodelling varies, with ventricular wall and septum thickening, systolic and diastolic dysfunction, and decreased left ventricular ejection fraction. Cardiac remodelling has an extremely complicated pathological mechanism, and different types of remodelling and pathological stages are regulated by distinct signalling pathways (Ritterhoff and Tian, 2023). Therefore, cardiac remodelling, accompanied by the pathological progression of infarction arrhythmias and chronic heart failure, is a risk factor for the development of many cardiovascular diseases and death (Chen et al., 2021). Although cardiac metabolic remodelling, cardiomyocyte apoptosis, phenotypic switching of cardiac fibroblasts, noncoding RNAs, immune regulation, protein translation, and epigenetic modifications have been reported to be associated with pathological cardiac remodelling, the mechanism is still unclear (Nakamura and Sadoshima, 2018). Clinically, angiotensin-converting enzyme/receptor inhibitors (ACEIs/ARBs), beta receptor blockers, and calcium channel blockers (CCBs) are commonly used to treat cardiac remodelling, however, these drugs aim to eliminate risk factors and relieve clinical symptoms, not fundamentally reverse or prevent the development of cardiac remodelling (Zhou et al., 2022). Therefore, screening and identifying new factors that play key regulatory roles in developing cardiac remodelling and elucidating their mechanisms are important for understanding the pathogenesis and identifying potential intervention targets for cardiac remodelling.

*Astragalus membranaceus* (Fisch.) Bunge root has long been used as an herbal Chinese medicine to protect against cardiovascular diseases (Liu et al., 2024). Various chemical constituents from *Astragalus membranaceus*, including triterpenoid saponins, flavonoids, and glycosides, have been reported in previous studies, however, it has still been challenging to elucidate the mechanism by which *Astragalus membranaceus* protects against cardiovascular diseases (Guo et al., 2019). When jointing analysis of serum proteomic profile in patients with heart failure and targets of *Astragalus membranaceus*, jaranol showed a potential biological activity for cardiac remodelling based on its 6 targets related to cardiac remodelling and good drug-like properties through Lipinski’s rule of five. Jaranol, also called as kumatakenin (5,4′-dihydroxy-3,7-dimethoxyflavone), is a flavonoid widely distributed in various Chinese herbal medicines, such as *liquorice (Glycyrrhiza* spp.*), Psychotria serpens* and *Siparuna cristata* (Xue et al., 2013; Chen, 2011). Although jaranol has been reported to have anticolitic (Arenbaoligao et al., 2023), anticancer (Woo et al., 2017), antiviral and microbial (Leal et al., 2021; Zhang et al., 2019), alpha-glucosidase inhibitory (Dao et al., 2021), and antioxidative (Quek et al., 2021) bioactivities, there have been no reports on the effects and mechanisms of jaranol in cardiovascular disease. The present study aimed to assess the beneficial effects of jaranol against pathological cardiac remodelling.

## Materials and methods

### Patients

Twenty patients were aged 18 years or older, had chronic heart failure had chronic heart failure (New York Heart Association functional class II, III, or IV) with a decreased left ventricular ejection fraction (≤50%), a significant change in cardiac structure, and a serum pro-B-type natriuretic peptide (NT-proBNP) level>2,500 pg/mL. The exclusion criteria were: (1) congenital heart disease, (2) acute coronary syndrome in the last 30 days, (3) pericardial disease, (4) tumors, and (5) metabolic diseases except for diabetes mellitus. Normal subjects were matched 1:1 according to the age and sex of the patients as controls. Serum proteomics analysis of 40 participants was performed to screen protein targets related to cardiac remodelling by APPLIED PROTEIN TECHNOLOGY Co., Ltd. (Shanghai, China). The study protocol was reviewed and approved by the Ethics Committee of Inner Mongolia Medical University (NO. YKD2019070). All participants gave written informed consent.

### Animal study

All the mice were housed in an environmentally controlled animal facility at Inner Mongolia Medical University with a room temperature of 22 °C–23 °C, a 12 h light/dark cycle, and access to a normal diet and purified water *ad libitum*. The mice were randomly divided into groups and acclimatized to the laboratory environments for at least 7 days before the experiments.

### Mouse cardiac remodelling model

A mouse model of cardiac remodelling was generated via transverse aortic constriction (TAC). The details of the TAC for the model and sham surgeries were described previously (Tavakoli et al., 2017). In brief, the mice were anaesthetized via an intraperitoneal injection of a single dose of 2% 2,2,2-tribromoethanol (Avertin) (Sigma‒Aldrich, St. Louis, MO). A 10 mm longitudinal midline cervical incision was made from the suprasternal notch to the mid-chest to expose the sternum after the fur in the surgical region was shaved and the skin was disinfected with 70% alcohol. The pretracheal muscles were separated to uncover the trachea, and then the pleura was removed. A 3–4 mm upper partial sternotomy was performed, and the aortic arch was located under low-power magnification. The aortic arch and a blunted 27-gauge needle were ligatured with a 6–0 suture line, after which the needle was removed quickly but gently to achieve 0.4 mm diameter narrowing and reproducible transverse 65%–70% aortic constriction. The sternum was sutured, and the skin was closed with a 5–0 suture line in one layer. The mice were allowed to warm until they were fully awake and were fed in standard cages. The sham mice underwent the same operation, but the aortic arch was not constricted.

### Jaranol treatment

Jaranol (221210X, purity >98%) was purchased from DASF Biotechnology Co., Ltd. (Nanjing, China); the chromatogram, LC/MS spectrum, and nuclear magnetic resonance (NMR) spectrum are shown in Supplementary Figures S1-S3. Jaranol was mixed with 0.1% carboxymethylcellulose sodium (CMC-Na), and the mixture was then sonicated for 3 min to prepare a suspension. Each animal received 50 mg/kg jaranol (300 μL) daily by gavage (Liu et al., 2022). The vehicle and model group mice received 300 μL of 0.1% CMC-Na daily by gavage. The mice were treated for 2 weeks before TAC and for 4 weeks after TAC.

### Jaranol targets and functional enrichment analysis

The SwissTargetPrediction Database was used to predict the most likely protein targets of jaranol on the basis of the similarity of the two-dimensional and three-dimensional chemical structures (Daina et al., 2019). Kyoto Encyclopedia of Genes and Genomes (KEGG) pathway analysis of jaranol targets was performed using the DAVID database (Dennis et al., 2003).

### Measurement of jaranol content in *Astragalus membranaceus*

#### Preparation of the control solution

A total of 1.03 mg of the jaranol standard was weighed accurately, and methanol was used to generate a standard solution with a concentration of 0.103 mg/mL; the solution was filtered through a 0.22 μm microporous membrane and stored as a stock solution. A gradient dilution of the stock solution was tested to establish standard curves and evaluate the sensitivity of the assay.

#### Preparation of the test solution

Approximately 10 g of crude powder of *Astragalus membranaceus* passed through a 10-mesh sieve was added to 100 mL of 50% ethanol. After soaking for 1 h, the mixture was heated and reflux extracted 2 times, each for 1 h, concentrated to 50 mL, and filtered through a 0.22 microporous filter membrane. The content of jaranol in *Astragalus membranaceus* was measured via HPLC.

### Echocardiography

An ultrasound diagnostic instrument (VINNO 10, Suzhou, China) was used to evaluate cardiac structure and function after TAC for 4 weeks. Briefly, the mice were first anesthetized with 3.0% isoflurane mixed with oxygen (100%, airflow velocity: 1 L/min). After the heart rate stabilized at 400 to 500 beats per minute after the pain reflex disappeared, the M-mode cursor was positioned perpendicular to the maximum LV dimension in end-diastole and systole, and M-mode images were obtained to measure wall thickness and chamber dimensions. Then, the ejection fraction and fractional shortening were automatically calculated by the software to evaluate cardiac structure and function.

### Cell culture, AngII stimulation, and jaranol treatment

#### Isolation and culture of primary neonatal mouse cardiomyocytes (CMs) and cardiac fibroblasts (CFs)

Primary cardiomyocytes and fibroblasts from neonatal mice were prepared using a Pierce Primary Cardiomyocyte Isolation Kit (88281; Thermo Fisher Scientific, USA). In brief, the ventricles of neonatal mice were cut into small pieces (approximately 1 mm^3^) and washed twice with ice-cold HBSS. A total of 0.2 mL of reconstituted cardiomyocyte isolation enzyme 1 (papain) and 10 µL of cardiomyocyte isolation enzyme 2 (thermolysin) were added to each tube. The contents of the tube were mixed gently and then incubated in a 37 °C incubator for 30–35 min. The enzyme mixture was gently removed, and the heart tissue was washed twice with 500 µL of ice-cold HBSS. Complete DMEM was added to each tube, and then the tissue was dissociated by pipetting up and down 25–30 times using a sterile 1.0 mL pipette tip to prepare a single-cell suspension. A total of 1.0 mL of complete DMEM was added to each tube to bring the total volume to 1.5 mL. The cells were counted after individual cell suspensions were combined and then seeded in 10 cm dishes. The dissociated cells seeded in 10 cm dishes were allowed to settle for 120 min to enrich cells on the basis of the differential adherence times of cardiac myocytes and fibroblasts. After being cultured for 120 min, the adherent cells were CFs, which were cultured continuously in a 10 cm dish with fresh Dulbecco’s modified Eagle’s medium (DMEM)/high-glucose supplemented with 10% FBS (11965092, Gibco). The cells in suspension were CMs, which were collected by centrifugation at 800 rpm for 5 min. After centrifugation, the CMs were resuspended in DMEM/high-glucose with 10% FBS and 0.1 mmol/L bromodeoxyuridine and then seeded in a 12- or 6-well plate at 2000 cells/cm^2^. The cells were maintained in an incubator at 37 °C in the presence of 5% CO_2_.

#### hiPSC-CM culture

Human induced pluripotent stem cell-derived cardiomyocytes (hPSC-CMs, CSM-H230011) were purchased from Cosmos Biotech Co., Ltd. (Nanjing, China) and resuscitated and cultured in a specialized medium according to the manufacturer’s instructions.

#### Cell experiment protocol

CMs, CFs, and *hiPSC-CMs* were randomly assigned to one of three experimental groups as follows: (1) control; (2) Ang II: 10^−6^ M angiotensin II; and (3) Ang II + jaranol: 10^−6^ M Ang II with 5 μM jaranol. At the beginning of the experiment, the CMs and CFs in the jaranol groups were pretreated with 5 μM jaranol in high-glucose FBS-free DMEM for 2 h, whereas those in the control and AngII groups were pretreated with high-glucose FBS-free DMEM for 2 h. After pretreatment, the culture medium was then replaced with fresh high-glucose FBS-free DMEM containing the appropriate drugs, and the cells were exposed to those drugs throughout the experimental period.

#### Culture and treatment of H9c2 cardiomyoblasts

Rat H9c2 cardiomyoblasts (ZQ0102, Zhong Qiao Xin Zhou Biotechnology, China) were cultured in 96-well plate within high glucose dulbeccos modified eagles medium (DMEM, 4.5 g/L glucose) that include penicillin (100 U/mL), streptomycin (100 μg/mL) and 10% fetal bovine serum for 24 h in a humidified atmosphere of 5% CO_2_ and 95% O_2_ at 37 °C. The H9c2 cardiomyoblasts were starved with no serum media for 24 h, then treated with 200, 100, 50, 25, 12.5, 6.25, 3.12, 1.56 μM of target compounds for 30 min, followed by treated with or without 10^−6^ M AngII for 24 h, the cell viability was detected by CCK-8 array.

### Cell counting kit-8 (CCK-8) assay

In brief, cells were incubated with 10% CCK-8 solution (96992, Sigma-Aldrich) at 37 °C for 1 h, and A450 was measured using a microplate reader (Thermo Fisher Scientific).

### Histological analysis

For histological analysis, the mice were first anaesthetized with 3.0% isoflurane mixed with oxygen (100%, airflow velocity: 1 L/min), and the hearts were arrested with a 10% potassium chloride solution at end-diastole and then fixed in 4% paraformaldehyde for 48 h. The fixed hearts were embedded in paraffin and cut transversely into 5 μm sections. Serial heart sections were stained with wheat germ agglutinin (WGA) (W11261, Invitrogen), and 100∼200 myocytes of left ventricle were outlined in each group to assess myocyte cross-sectional areas. The degree of collagen deposition was evaluated by picrosirius red (PSR) staining (ab150681, Abcam), and 10∼20 fields of left ventricle were outlined in each heart. The images were analysed using a quantitative digital image analysis system (Image-Pro Plus 6.0) in a blinded manner.

### CM and hiPSC-CM phalloidin staining

CMs and hiPSC-CMs were cultured in high glucose FBS-free DMEM containing different drugs for 48 h and then were stained with iFluor™ 488-labelled phalloidin (40736ES75, Yeasen Biotechnology, Shanghai, China) to evaluate myocyte size under a fluorescence microscope (Zeiss, Germany). In brief, the cells were washed twice with prewarmed phosphate-buffered saline (pH 7.4) and then fixed in a 3.7% formaldehyde solution for 10 min at room temperature. The cells were washed twice with PBS and incubated with 0.1% Triton X-100 in PBS for 5 min. After the cells were washed twice with PBS, they were stained with 200 μL of phalloidin at room temperature for 30 min. The cells were washed twice with PBS, and nuclei were stained with 4,6-diamidino-2-phenylindole (DAPI, H-1200, Vector Laboratories). Images were obtained using a laser scanning confocal microscope (Carl Zeiss Inc., Thornwood, NY, USA) at excitation/emission wavelengths of 493/517 nm and E364/454 nm (DAPI). Fluorescence was quantified using Image-Pro 6.0 (Media Cybernetics Inc., Bethesda, MD, USA).

### Western blotting and quantitative real-time PCR

Proteins were extracted from mouse hearts with radioimmunoprecipitation assay (RIPA) buffer (50 mM Tris-HCl pH 7.4, 150 mM NaCl, 1 mM EDTA, 0.25% sodium deoxycholate, 0.1% SDS and protease inhibitor cocktail). Homogenates were sonicated and centrifuged at 4 °C for 15 min, and the supernatants were used for Western blotting. Nuclear and cytoplasmic proteins were separated from CMs using a Minute Cytoplasmic and Nuclear Extraction Kit (sc003, Invent Biotechnologies). For each sample, 20–50 μg of protein was separated by SDS‒PAGE and transferred to an NC membrane (HATF00010, Millipore). After being blocked with 5% nonfat milk, the membrane was incubated with primary antibodies overnight at 4 °C. The membrane was subsequently incubated with a horseradish peroxidase-conjugated secondary antibody (1:10000; sc-2357; Santa Cruz Biotechnology) and exposed to an enhanced chemiluminescence (ECL) reagent to detect protein bands.

Total RNA from mouse hearts, CMs, and CFs was extracted using TRIzol reagent (15596026, Invitrogen), and first-strand cDNA was synthesized using reverse transcriptase (3735A, Takara). Real-time PCR was performed with SYBR Green to examine the relative mRNA levels of the indicated genes. The sequences of the real-time PCR primers are shown in Supplementary Table S1.

### TLR2 expression assessment in CMs

CMs were cultured in 12-well plate in high glucose FBS-free DMEM, and randomly assigned to one of two experimental groups as follows: (1) control; (2) Snai3: 0.18 μM Snai3. CMs were treated with or without 0.18 μM Snai3 in high-glucose FBS-free DMEM for 48 h, then TLR2 expression in CMs was detected by Western blot.

### RNA sequencing

Total RNA from the mouse left ventricles in the different treatment groups was subjected to transcriptome sequencing by APPLIED PROTEIN TECHNOLOGY Co., Ltd. (Shanghai, China). Five biological replicates were performed for each group. Functional enrichment analyses of differentially expressed genes were performed using R software (version 4.2).

### Adeno-associated virus vectors and vector delivery *in vivo*

Adeno-associated virus 9 vectors expressing human TLR2 (Ad-TLR2) and GFP(Ad-GFP) were constructed by Vigene Biosciences, Inc. (Shandong, China). The average viral titres were 9.23 × 10^13^ and 7.46 × 10^13^ vector genome (VG) particles/mL for the Ad-TLR2 and Ad-GFP vectors, respectively. Vectors were diluted with sterile saline, and 100 µL of 5 × 10^11^ VG/mouse Ad-TLR2 vector was intravenously injected into the mouse tail vein using a 27G insulin syringe (BD Biosciences, Franklin Lakes, NJ, USA); the same dose of the Ad-GFP vector was injected into the control group.

After receiving vector injections for 3 weeks, the jaranol treatment group received 50 mg/kg jaranol (300 μL) per day by gavage, and the control group received 300 μL 0.1% CMC-Na orally per day. After 2 weeks of jaranol treatment, cardiac hypertrophy was induced by TAC for 4 weeks. At the end of the experiment, echocardiography and histological analysis were performed to evaluate heart function and the degree of cardiac remodelling.

### Molecular docking

The chemical structure of jaranol was obtained through a PubMed search, and the planar structure was drawn in ChemDraw 21.0.0, converted into a 3D configuration using Chem3D 21.0.0 and exported in MOL format. Then, the compound structure was processed in AutoDock Tools 1.5.6 (hydrogenation, ligand setting, and torsion bond detection) and exported in PDBQT format. The structure of NF-κB was obtained from the RCSB database and was subjected to preprocessing with AutoDock Tools 1.5.6 (addition of hydrogen, assignment of partial charges and protonation states, and removal of water molecules and primitive ligands). The resulting data were exported in PDBQT format. The original location of the protein ligand was used as the ligand space to generate the config. txt file after setting up the docking box. Subsequently, QuickVina-W was run on Linux to dock the processed ligand and receptor PDBQT files as well as the comfig. txt file, and the docking results were visualized in PyMoL 2.6 and AutoDock Tools 1.5.6.

### Proteome microarray assays

Arrayit HuProt™ v2.0 19K Human Proteome Microarrays (CDI Laboratories, Baltimore, MD) were used to identify jaranol-interacting proteins. The microarrays were blocked with blocking buffer for 1.5 h at room temperature and protected from light. After blocking, the blocking buffer was discarded, and the chip was washed once with 1× PBS-T for 5 min. The chip was cultured with 50 µM biotinylated-jaranol or free biotin at room temperature for 1 h. After culturing, the chip was washed once with 1× PBS-T for 5 min and then with deionized water twice (5 min each). After washing, Cy5-streptavidn solution (1:1000, Sigma‒Aldrich, St. Louis, MO) was added. GenePix 4000B (Axon Instruments, Sunnyvale, CA) was used for scanning. The signal-to-noise ratio was defined as the ratio of the foreground and background values. To call candidates, the cut-off was set at a ratio >3.0. The data were analysed using GenePix Pro 6.0. The entire experimental protocol, including the synthesis of biotinylated jaranol and proteome microarray assays, was performed at Wayenbiotech Co., Ltd. (Shanghai, China).

### Subcellular fractionation of hiPSC-CM

Cytoplasmic and nuclear extracts were prepared according to the instructions of the NE-PER® nuclear and cytoplasmic extraction kit (Pierce).

### Statistical analysis

All the data are presented as means ± standard deviation (SD). One-way or two-way analysis of variance (ANOVA) followed by Tukey’s *post hoc* test was used for statistical comparisons among more than two groups. Statistical analyses were performed with GraphPad Prism 8.0 (GraphPad Software, Inc.). The “WGCNA” package was used to construct the weighted protein coexpression network. *P* < 0.05 indicated a statistically significant difference.

## Results

### Joint analysis indicated jaranol with potential activity against cardiac remodelling

**FIGURE 1:**
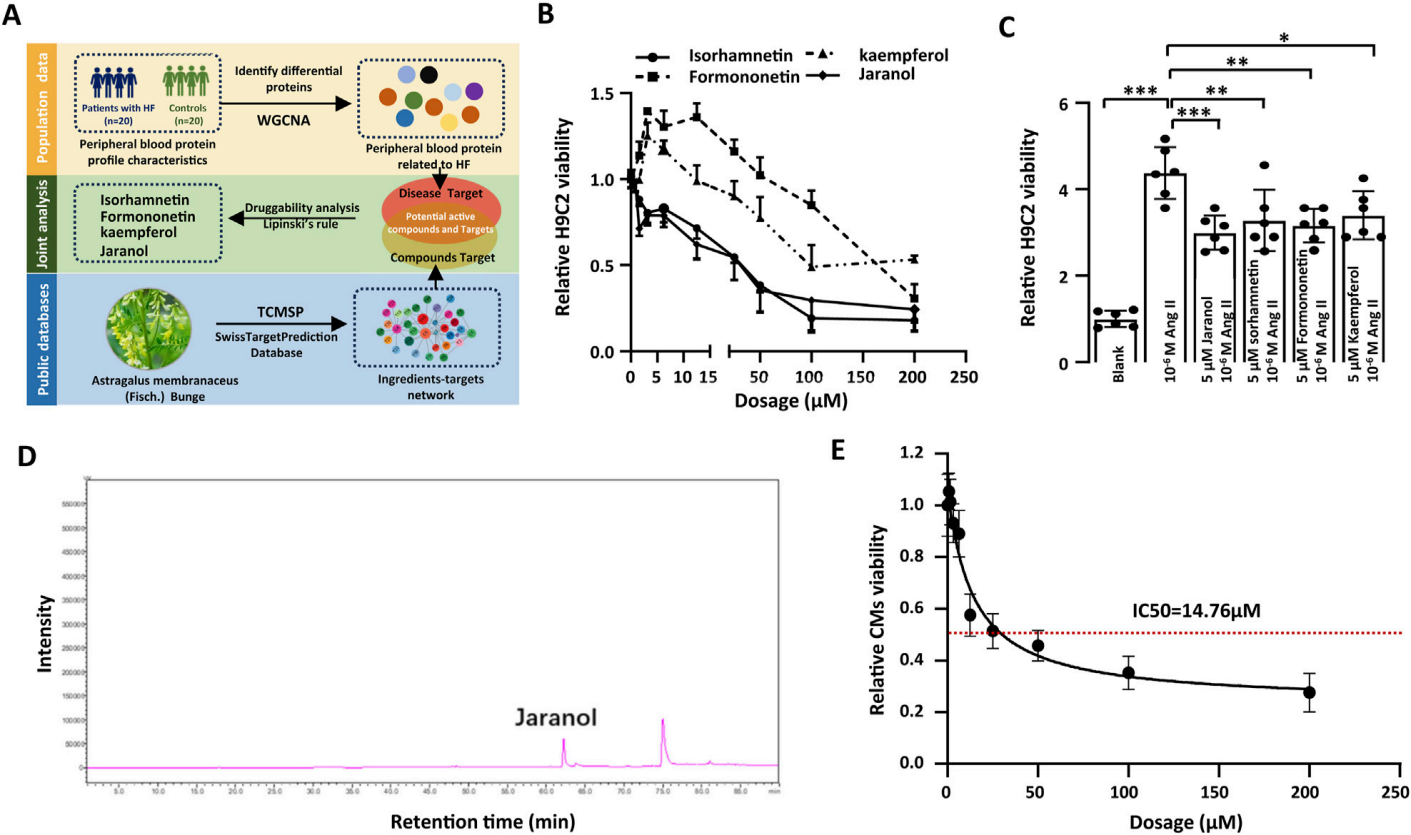
Joint analysis indicated jaranol with potential activity against cardiac remodelling. **(A)** Schematic illustrating screening process of target compounds, WGCNA: Weighted Gene Co-expression Network Analysis, TCMSP: Traditional Chinese Medicine Systems Pharmacology Database and Analysis Platform; **(B)** Cell viability analyzed by CCK-8 array in H9c2 cardiomyoblast treated with target compounds at different doses for 24 h (n = 6 per group); **(C)** Cell viability analyzed by CCK-8 array in H9c2 cardiomyoblast treated with 5 μM target compounds and 10^−6^ M AngII for 24 h (n = 6 per group); **(D)** HPLC chromatogram of jaranol; **(E)** Cell viability analyzed by CCK-8 array in CMs treated with jaranol at different doses for 24 h (n = 6 per group). The data are presented as means±SDs; one-way ANOVA with Tukey’s post hoc test; ^*^ *P* < 0.05, ^**^ *P* < 0.01, and ^***^ *P* < 0.001. CMs: cardiomyocytes.

We investigated possible correlations between serum proteins and the phenotype of heart failure by weighted correlation network analysis (WGCNA) to screen potential targets related to cardiac remodelling. The baseline characteristics of patients with heart failure and controls are shown in Supplementary Table S2. The total of 672 proteins were considered as the potential targets of cardiac remodelling (Supplementary Figure S4). To screen active compounds, total of 1073 targets of 66 ingredients in Astragalus membranaceus were anticipated in Traditional Chinese Medicine Systems Pharmacology (TCMSP) platform and SwissTargetPrediction Databases (Supplementary Table S3). Finally, 23 ingredients in *Astragalus membranaceus* could regulate at least 5 targets related to cardiac remodlling among 66 ingredients by overlapping between targets of ingredients in *Astragalus membranaceus* and targets related to cardiac remodelling from WGCNA of serum proteomics. According to Lipinski’s rule of five, Jaranol, Isorhamnetin, Formononetin, and Kaempferol were considered to have the potential to be used as a drug (Supplementary Figure S5; Supplementary Table S4) (Lipinski, 2004). Schematic illustrating screening process was showed in Figure 1A.

The CCK-8 assay further confirmed low dosage of kaempferol and formononetin (<5 μM) promoted H9c2 viability, while jaranol and isorhamnetin continuously suppressed H9c2 viability (Figure 1B). Besides, four compounds significantly suppressed 10^−6^ M AngII-induced increase in H9c2 viability (Figure 1C). Meanwhile, the previous studies showed a clear protective effect of Isorhamnetin, Formononetin, and Kaempferol on multiple causes–induced cardiac remodelling, further validating the accuracy of our analytical method (Liu et al., 2017; Qian et al., 2024; Gao et al., 2017). Therefore, we decided to study the effect of jaranol on cardiac remodelling, and evaluate the possible mechanism *in vitro* and *in vivo*.

The biological analysis results showed that jaranol regulated multiple protein targets related to cardiac remodelling, and target function analysis revealed that some signalling pathways closely associated with cardiac remodelling were significantly enriched (Supplementary Table S5, Supplementary Figure S6). Based on the potential therapeutic effects of jaranol on cardiac remodelling, we first examined the content of jaranol in Astragalus membranaceus via high-pressure liquid chromatography (HPLC), and the results revealed that the average jaranol content was 0.045 μg/g in 16 batches of Astragalus membranaceus (Figure 1D; Supplementary Table S6). The CCK-8 assay revealed that the activity of mouse primary cardiomyocytes (CMs) decreased gradually with increasing jaranol concentration, ultimately, 5 μM jaranol was selected to evaluate its biological ability for AngII-induced cardiac remodelling *in vitro* (Figure 1E).

### Jaranol ameliorates AngII-induced cardiac remodelling *in vitro*

**FIGURE 2:**
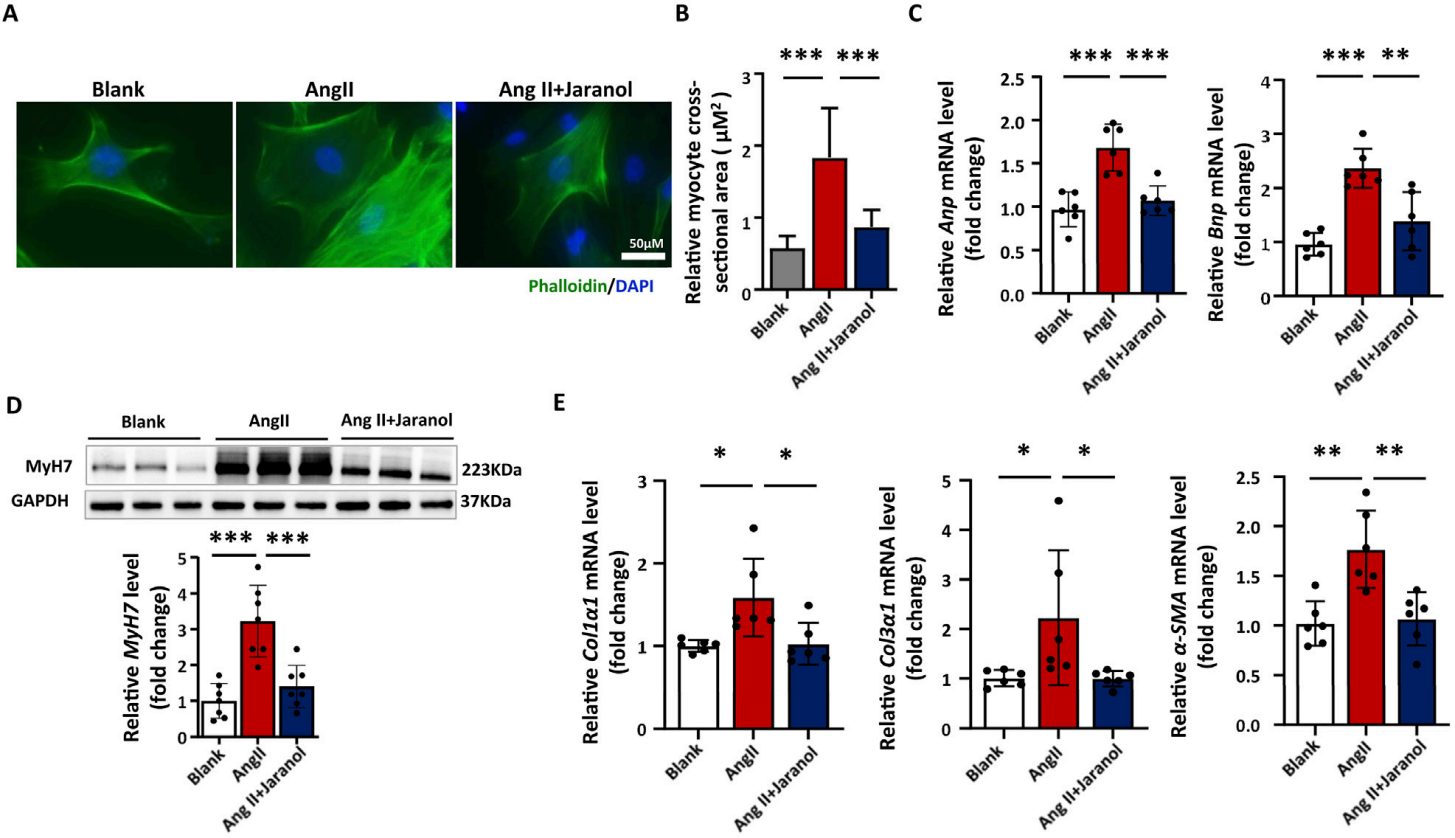
Jaranol ameliorates AngII-induced cardiac remodelling *in vitro*. **(A)** Immunofluorescence staining of neonatal CMs treated with or without jaranol during Ang II (angiotensin II)-induced hypertrophy. Scale bar = 50 µm. **(B)** Quantification of the size of CMs treated with or without jaranol during Ang II-induced hypertrophy (n = 6 per group). **(C)** RT‒qPCR analysis of Anp and Bnp mRNA levels in CMs treated with or without jaranol during Ang II-induced hypertrophy (n = 6 per group). **(D)** A representative Western blot image showing MyH7 protein levels in CMs, and quantification of MyH7 protein levels in CMs (n = 6 per group). **(E)** RT‒qPCR analysis of Col1α1, Col3α1, α-SMA mRNA levels in CFs treated with or without jaranol during Ang II-induced hypertrophy (n = 6 per group). The data are presented as means±SDs; one-way ANOVA with Tukey’s post hoc test; ^*^ *P* < 0.05, ^**^ *P* < 0.01, and ^***^ *P* < 0.001. CMs: cardiomyocytes; CFs: cardiac fibroblasts.

We found that Ang II induced an increase in cell size for 48 h (Figures 2A,B), and an increase in the expression of cardiac remodelling-related genes (Anp and Bnp) for 24 h in CMs (Figure 2C); these effects were significantly inhibited in jaranol-treated CMs. The expression level of MYH7 as the hypertrophic marker significantly increased in Ang II-induced CMs for 48 h, which was inhibited in jaranol-treated CMs (Figure 2D). In CFs, jaranol treatment for 24 h significantly inhibited the AngII-induced expression of phenotype shift-related genes (Col1α1, Col3α1, and α-SMA) (Figure 2E). Collectively, these data suggested that jaranol had a protective effect against Ang II-induced cardiac remodelling *in vitro*.

### Jaranol ameliorates TAC-induced cardiac remodelling *in vivo*

**FIGURE 3:**
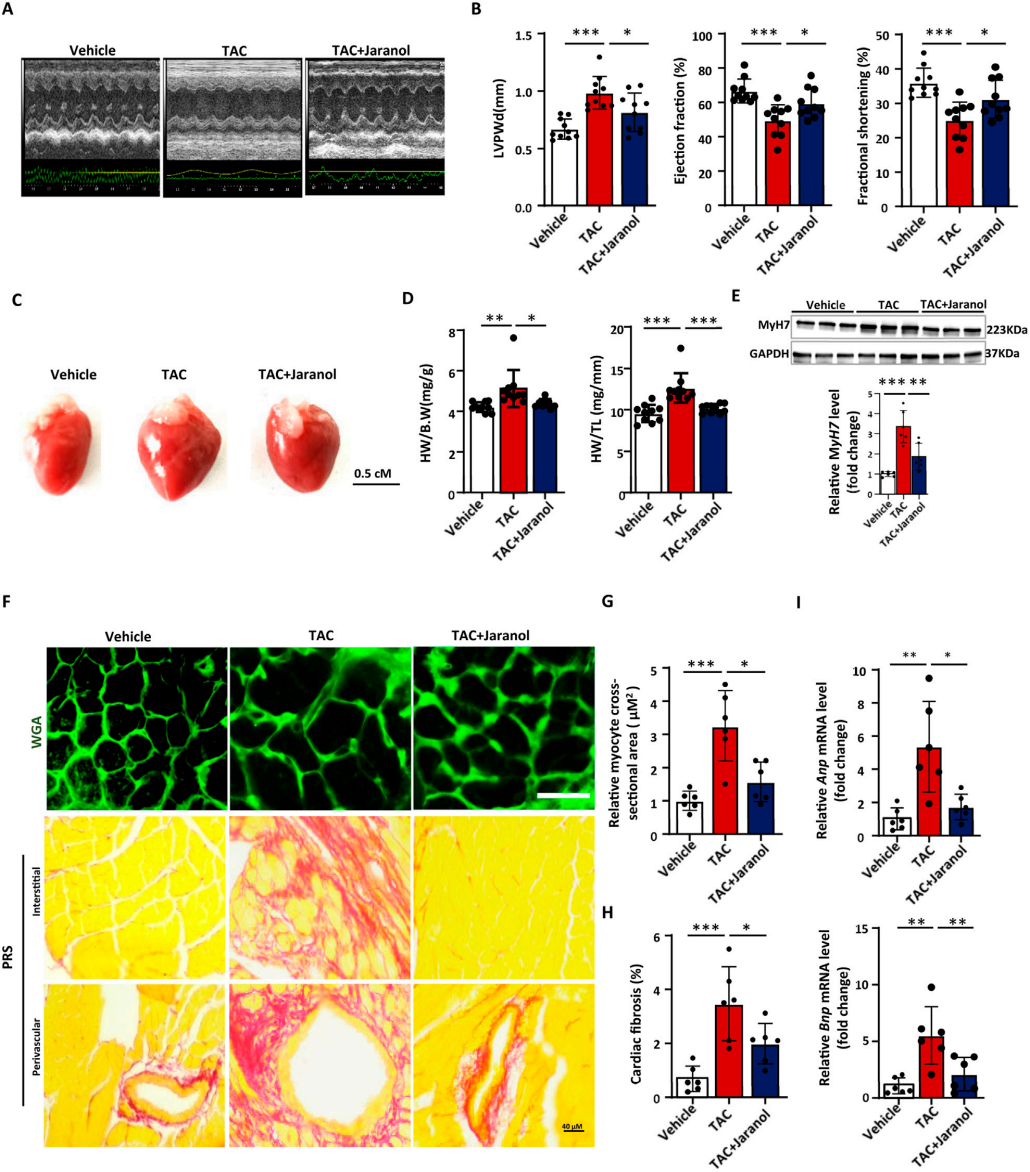
Jaranol ameliorates TAC-induced cardiac remodelling *in vivo.* **(A)** Representative echocardiographic images of the mice in each group. **(B)** Cardiac structural and functional tests showing the LVPWd, EF, and FS (n = 10 per group). **(C)** Representative gross appearance of whole hearts; scale bar = 0.5 cm. **(D)** The ratio of heart weight to body weight (HW/BW) and tibia length (HW/TL) (n = 10 per group). **(E)** A representative Western blot image showing MyH7 protein levels in mouse heart, and quantification of MyH7 protein levels in mouse heart (n = 6 per group). **(F)** Heart tissues were stained with fluorophore-conjugated wheat germ agglutinin (WGA) and picrosirius red staining to assess cardiomyocyte hypertrophy (Scale bar = 20 µm) and cardiac fibrosis (Scale bar = 40 µm); **(G)** Statistical results for the cell cross-sectional area (n = 6 per group). **(H)** Quantification of the interstitial fibrotic area (%) in Sirius Red-stained heart sections (n = 6 per group). **(I)** RT‒qPCR analysis of cardiomyocyte hypertrophic marker (Anp, Bnp) mRNA levels (n = 6 per group). The data are presented as the means±SDs; one-way ANOVA with Tukey’s post hoc test; ^*^ *P* < 0.05, ^**^ *P* < 0.01, and ^***^ *P* < 0.001.

To further investigate the protective effect of jaranol against cardiac remodelling, adult wild-type mice were subjected to TAC, a surgical model of pressure overload-induced cardiac remodelling. As expected, compared with mice in the vehicle group, mice in the TAC group presented changes in cardiac structure, systolic and diastolic function, as illustrated by an increase in the left ventricular posterior wall thickness at end diastole (LVPWd) and a decrease in the ejection fraction (EF) and fraction shortening (FS) in the heart; however, these effects were reversed in the hearts of jaranol-treated mice (Figures 3A,B; Supplementary Table S7). In addition, mice in the jaranol-treated group presented a significantly ameliorated phenotype, with a reduced heart size and heart weight compared with mice in the TAC group (Figures 3C,D). The expression level of MYH7 as the hypertrophic marker significantly increased in heart of TAC-induced mice compared to vehicle mice for 4 weeks, which was inhibited in heart of jaranol-treated mice compared to TAC-induced mice (Figure 3E). Cardiac sections stained with WGA revealed that, compared with mice in the vehicle group, mice in the TAC group presented marked cardiac hypertrophy 4 weeks after surgery, as revealed by an increased cardiomyocyte cross-sectional area. Compared with mice in the TAC group, mice in the jaranol-treatment group exhibited a decreased response to TAC-induced cardiac hypertrophy (Figures 3F,G). Sirius red staining showed that, compared with mice in the vehicle group, mice in the TAC group presented significantly greater fibrosis in the interstitial and perivascular areas of the heart, and jaranol treatment for 4 weeks alleviated TAC-induced cardiac fibrosis (Figures 3F,H). In addition, the expression of cardiac remodelling-related genes (Anp and Bnp) was significantly greater in the hearts of mice in the TAC group than in the hearts of mice in the vehicle group 4 weeks after surgery, whereas the expression of these genes was lower in the hearts of mice in the jaranol-treatment group than in the hearts of mice in the TAC group (Figure 3I). Overall, jaranol treatment ameliorated TAC-induced cardiac remodelling *in vivo.*

### The protective effect of jaranol against cardiac remodelling is associated with suppressing TLR signalling pathway

**FIGURE 4:**
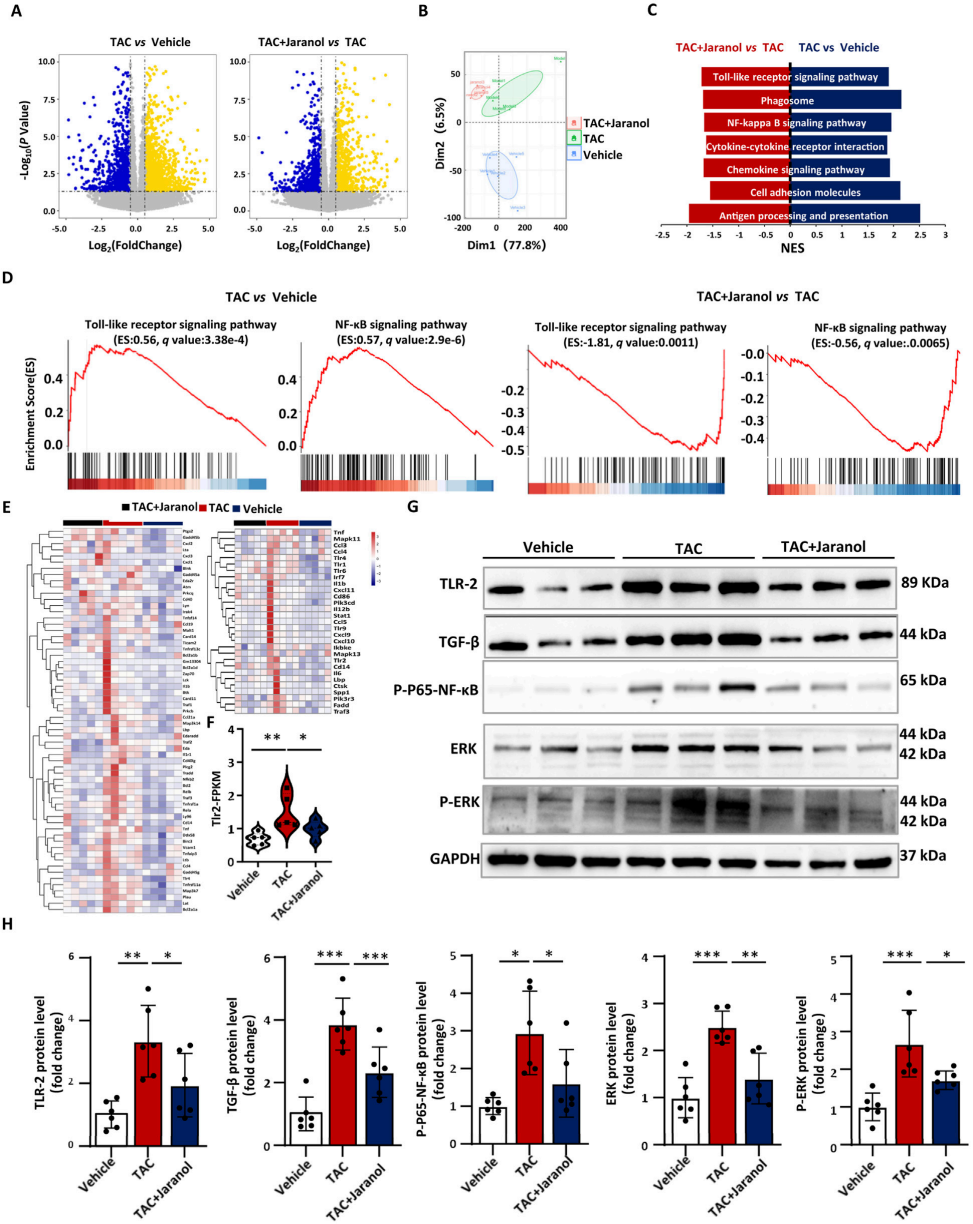
The protective effect of jaranol against cardiac remodelling is associated with the regulation of the TLR signalling pathway. **(A)** Volcano plots showing changes in cardiac gene expression in different mice; genes whose expression significantly increased (up) and decreased (down) are highlighted in yellow and blue, respectively. **(B)** Principal component analysis (PCA) plot of genes expression in mouse heart from different groups (n = 5 per group), elliptical areas represent 95% confidence regions. **(C)**Gene set enrichment analysis of genes whose expression significantly increased (up) or decreased (down) in mouse hearts was performed to evaluate the mechanism of jaranol action. **(D)** Gene set enrichment analysis plots of the Toll-like receptor signalling pathway and NF-κB signalling pathway in different mouse hearts 4 weeks after TAC. NES, normalized enrichment score. **(E)** Heatmap showing the expression levels of Toll-like receptor and NF-κB signalling pathway-related genes. **(F)** The FPKM value of Tlr2 in different groups (n = 5 per group). **(G,H)** Western blot analysis and densitometric quantification of TLR-2, TGF-β, P-P65-NF-κB, ERK, and P-ERK in different mouse hearts, with GAPDH as a control (n = 6 per group). The data are presented as means±SDs; one-way ANOVA with Tukey’s post hoc test; ^*^ *P* < 0.05, ^**^ *P* < 0.01, and ^***^ *P* < 0.001.

To investigate the underlying mechanism by which jaranol alleviated cardiac remodelling, RNA sequencing of mouse hearts was performed, with five replicates per group. Genes with│log_2_ Foldchange│≥0.58 and P < 0.05 were considered differentially expressed genes (DEGs) between groups. Compared with those in the hearts of mice in the vehicle group, 1210 and 575 genes were significantly upregulated and downregulated, respectively, in the hearts of mice in the TAC group. Compared with that in the hearts of mice in the TAC group, the expression of 671 and 517 genes in the hearts of jaranol-treated mice was significantly upregulated and downregulated, respectively (Figure 4A). Principal component analysis (PCA) showed a clear difference among different groups, indicating that gene expression characteristics in mouse heart of jaranol-treated mice exhibited different from vehicle and model mice (Figure 4B). Gene set enrichment analysis of the DEGs revealed that jaranol treatment inhibited several signalling pathways related to cardiac remodelling in mouse hearts, such as the NF-κB signalling pathway (Takemoto et al., 1999), cell adhesion molecules (Liu et al., 2019), and the chemokine signalling pathway (Wang et al., 2018) (Figure 4C). Further analysis revealed that the Toll-like receptor signalling pathway and NF-κB signalling pathway were significantly more active in the hearts of mice in the TAC group than in the hearts of mice in the vehicle group and was suppressed in the hearts of mice in the jaranol-treatment group relative to that observed in the hearts of mice in the TAC group (Figures 4D,E). Among differential expression genes among groups, Tlr2 gene was significantly upregulated in heart of TAC-induced mice compared with vehicle mice, however, which was significantly downregulated in heart of jaranol-treated mice compared with TAC-induced mice (Figure 4F). To validate the RNA sequencing results, the expression levels of TLR2, TGF-β, P-P65-NF-κB, ERK, and p-ERK in mouse hearts were examined by Western blotting. The expression levels of these proteins were significantly higher in the hearts of mice in the TAC group than in the hearts of mice in the vehicle group 4 weeks after surgery, and jaranol treatment inhibited the TAC-induced expression of these proteins in the heart (Figures 4G,H). Previous studies have shown that cardiac-specific TLR2 overexpression aggravates cardiac remodelling, whereas the inhibition of TLR2 improves cardiac pathological remodelling (Wang et al., 2014). Therefore, we speculated that the protective effect of jaranol against cardiac remodelling was related to the inhibition of TAC-induced TLR2 expression in the heart.

### Cardiac-specific TLR2 overexpression exacerbated TAC-induced cardiac remodelling and reversed the protective effect of jaranol against cardiac remodelling

**FIGURE 5:**
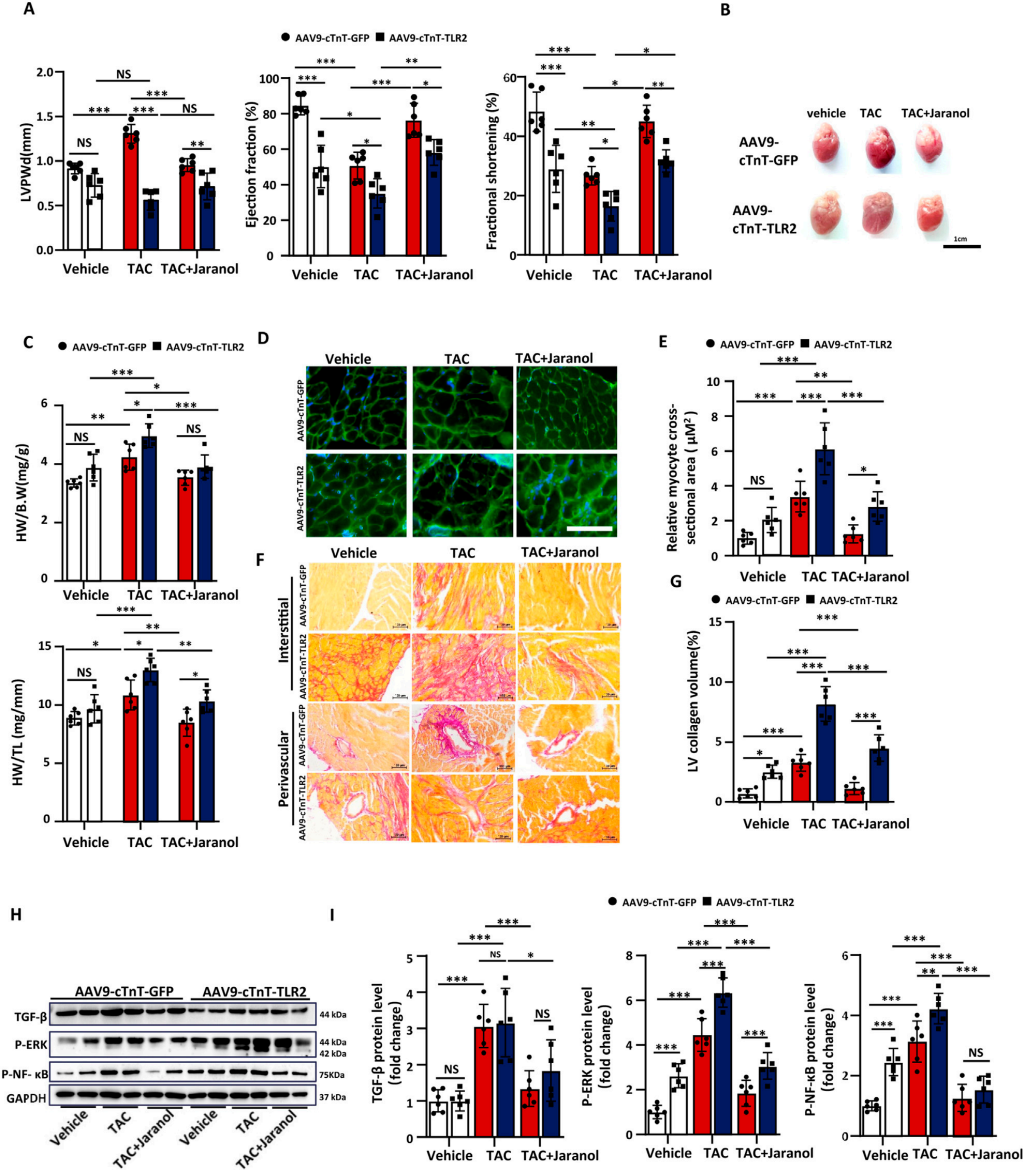
Cardiac-specific TLR2 overexpression exacerbated TAC-induced cardiac remodelling and reversed the protective effect of jaranol against cardiac remodelling. **(A)**Cardiac structural and functional tests showing the LVPWd, EF, and FS from AAV9-cTnT-GFP- and AAV9-cTnT-TLR2-injected mice 4 weeks after TAC or vehicle surgery (n = 6 per group). **(B)** Representative images of the gross morphology of hearts from AAV9-cTnT-GFP- and AAV9-cTnT-TLR2-injected mice 4 weeks after vehicle or TAC surgery. Scale bar = 1 cm. **(C)** The ratio of heart weight to body weight (HW/B.W) and tibia length (HW/TL) for AAV9-cTnT-GFP- and AAV9-cTnT-TLR2-injected mice 4 weeks after vehicle or TAC surgery (n = 6 per group). **(D)** Representative images of left ventricular muscle sections stained with WGA from mice in different groups. Scale bar = 40 µm. **(E)** Quantitative analysis of cross-sections of WGA-stained cardiomyocytes from AAV9-cTnT-GFP- and AAV9-cTnT-TLR2-injected mice 4 weeks after vehicle or TAC surgery (n = 6 per group). **(F)** Representative images of Picro Sirius Red-stained histological sections from AAV9-cTnT-GFP- and AAV9-cTnT-TLR2-injected mice 4 weeks after vehicle or TAC surgery; scale bar = 10 µm. **(G)** Quantitative analysis of left ventricle (LV) collagen content in AAV9-cTnT-GFP- and AAV9-cTnT-TLR2-injected mice (n = 6 per group). **(H,I)** Immunoblots and densitometric quantification showing the expression levels of TGF-β, NF-κB, and ERK, with GAPDH as a control, in myocardia from AAV9-cTnT-GFP- and AAV9-cTnT-TLR2-injected mice 4 weeks after vehicle or TAC surgery (n = 6 per group). The data are presented as means±SDs; two-way ANOVA with Tukey’s post hoc test; ^*^ *P* < 0.05, ^**^ *P* < 0.01, and ^***^ *P* < 0.001; NS: not significant.

To further understand the relationship between the protective effect of jaranol against cardiac remodelling and TLR2 signalling, we engineered an adeno-associated virus-9 (AAV-9) vector to overexpress TLR2 under the control of the cardiomyocyte-specific cardiac troponin (cTnT) promoter. The mice were injected with 10^12^ AAV9 viral particles 5 weeks before TAC surgery. Three weeks after virus injection, jaranol-treated mice received 50 mg/kg jaranol (300 μL) per day by gavage for 2 weeks. After 2 weeks of jaranol pretreatment, mice in the TAC group and jaranol-treatment group underwent TAC surgery, and cardiac function was analysed by echocardiography 4 weeks after surgery (Supplementary Figure S7). The results revealed that TLR2 expression was significantly higher in the hearts of AAV9-TLR2-injected mice than in AAV9-control-injected mice 3 weeks after injection (Supplementary Figure S8). Representative echocardiographic M-mode images are shown in Supplementary Figure S9. Cardiac-specific TLR2 overexpression significantly altered cardiac structure and function, as demonstrated by decreases in the LVPWd, EF, and FS in the hearts of AAV9-TLR2-injected mice. Four weeks after TAC surgery, AAV9-control-injected mice presented an increase in LVPWd and a decrease in EF and FS, which are typical phenotypes of hypertrophic cardiomyopathy. A significantly lower LVPWd in AAV9-TLR2-injected mice than in AAV9-control-injected mice repressed dilated cardiomyopathy; moreover, the cardiac-specific overexpression of TLR2 aggravated TAC-induced cardiac dysfunction. Jaranol treatment had a significant therapeutic effect on the cardiac structure and functional response to TAC for 4 weeks in both the AAV9-control- and AAV9-TLR2-injected mice, but these effects were reversed by cardiac-specific TLR2 overexpression (Figure 5A). Similarly, the effect of jaranol on morphological improvements was evidenced by a lower heart weight-to-body weight ratio and heart weight-to-tibial length ratio in mice in the jaranol-treatment group than in mice in the TAC group 4 weeks after TAC. Nevertheless, a significant increase in the heart weight-to-tibial length ratio in AAV9-TLR2-injected mice compared with that in AAV9-control-injected mice indicated that jaranol activity decreased due to TLR2 overexpression (Figures 5B,C). WGA staining of myocardial tissue revealed that the cross-sectional area of cardiomyocytes was significantly smaller in the hearts of mice in the jaranol-treatment group than in the hearts of mice in the TAC group, illustrating the protective effect of jaranol against TAC-induced cardiac hypertrophy; however, this effect was blunted after TLR2 overexpression (Figures 5D,E). Although there was no influence on the cross-sectional area of cardiomyocytes under normal physiological conditions, cardiac-specific TLR2 overexpression for 3 weeks not only led to fibrosis but also exacerbated TAC-induced cardiac fibrosis. Jaranol treatment significantly alleviated TAC-induced cardiac fibrosis in both the AAV9-control- and AAV9-TLR2-injected mice, but cardiac fibrosis levels were significantly greater in the hearts of the jaranol-treated AAV9-TLR2-injected mice than in those of the jaranol-treated AAV9-control-injected mice, which indicated a decreased protective effect of jaranol against TAC-induced cardiac fibrosis after TLR2 overexpression (Figures 5F,G). At the molecular level, cardiac-specific TLR2 overexpression promoted the TAC-induced expression of hypertrophic markers, such as atrial natriuretic peptide (Anp) and brain natriuretic peptide (Bnp), while it inhibited the effect of jaranol on the expression of hypertrophic markers (Supplementary Figure S10). At the protein level, cardiac-specific TLR2 overexpression did not affect TAC-induced TGF-β expression in the heart; however, it upregulated the expression levels of P-ERK and P-NF-κB in the hearts of AAV9-TLR2-injected mice compared with those in the hearts of AAV9-control-injected mice. In addition, the inhibitory effect of jaranol on TAC-induced P-ERK expression in the hearts of AAV9-TLR-2-injected mice was significantly attenuated compared with that in the hearts of AAV9-control-injected mice, but a similar result was not found for P-NF-κB expression (Figures 5H,I). These data demonstrated that cardiac-specific TLR2 overexpression exacerbated TAC-induced cardiac remodelling while reversing the protective effect of jaranol against cardiac remodelling.

### Transcription factor Snai3 was involved in TAC and jaranol-regulated TLR2 expression in mouse heart

**FIGURE 6:**
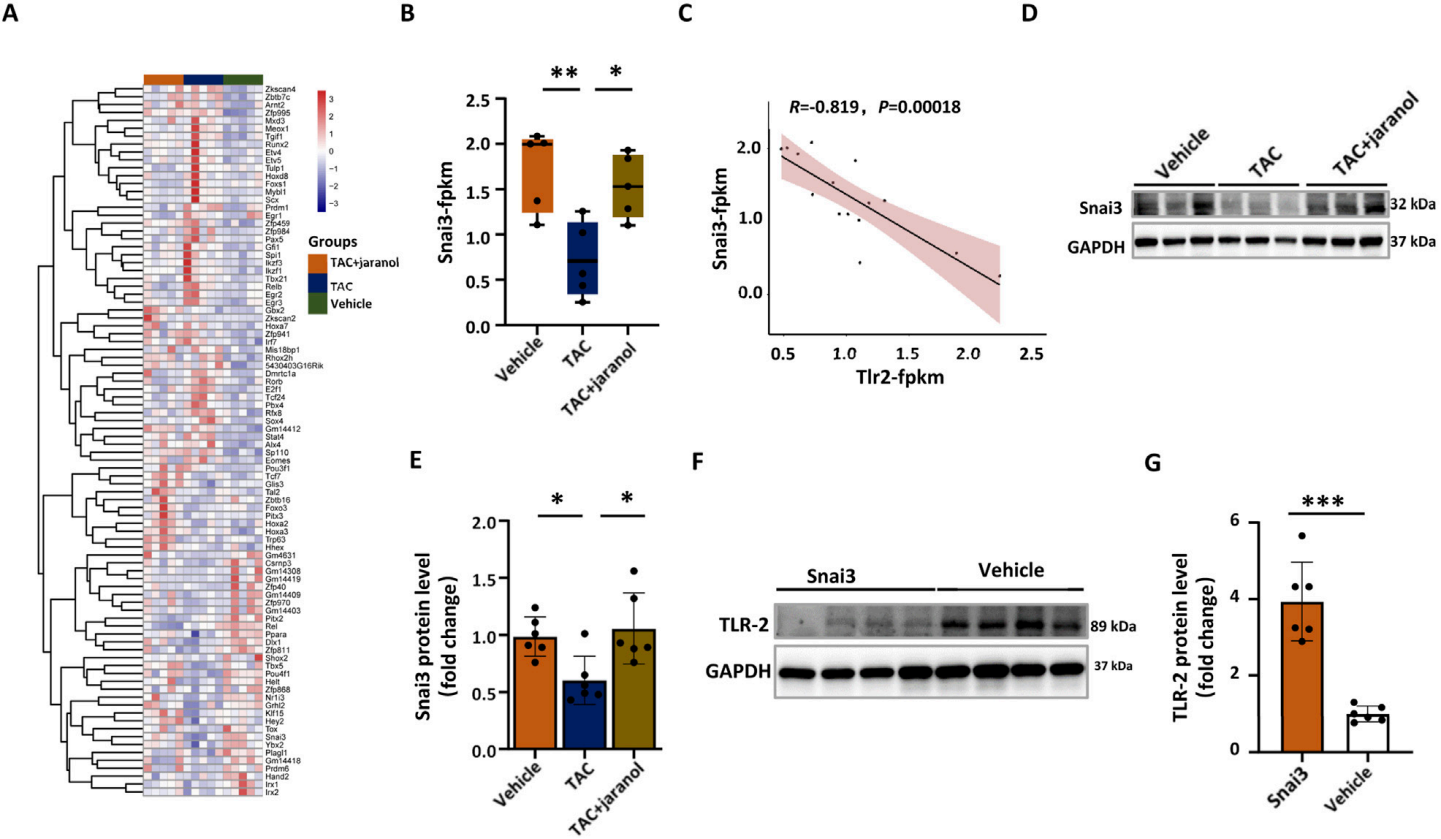
Transcription factor Snai3 was involved in TAC and jaranol-regulated TLR2 expression in mouse heart. **(A)** Heatmap of transcription factors with significantly differential expression in mouse heart between groups; **(B)** Snai3 expression level in different groups (n = 5 per group); **(C)** Significantly negative correlation between the Snai3 and TLR2 expression in mouse heart; **(D)** Western blot analysis of Snai3 protein levels in mouse heart tissues; **(E)** Quantification of the Western blot signals is shown as fold change compared with vehicle (n = 6 per group),the data are presented as means±SDs, one-way ANOVA with Tukey’s post hoc test; ^*^ *P* < 0.05; **(F)** Western blot analysis of Snai3 protein levels in CMs; **(G)** Quantification of the Western blot signals is shown as fold change compared with vehicle (n = 6 per group), the data are presented as means±SDs, Student’s t-test; ^***^ *P* < 0.001. CMs: cardiomyocytes.

Next, we investigated the molecular mechanism by which jaranol regulate TAC-induced TLR2 expression. The mRNA-sequencing of mouse myocardium showed that the expression of 90 transcription factors were significantly altered in mouse heart between different groups (Figure 6A). Among 90 transcription factors, Snai3 expression was significantly downregulated in heart of TAC-induced mice compared with vehicle mice, however, it was significantly upregulated in heart of jaranol-treated mice compared with TAC-induced mice (Figure 6B). Further analysis revealed a significant negative correlation between Snai3 and TLR2 expression in mouse heart (Figure 6C). Western blot result also showed jaranol administration inhibited TAC-induced decrease in Snai3 expression in mouse heart (Figures 6D,E). Therefore, we speculated that transcription factor Snai3 could bind to the promoter region of the TLR2 gene to inhibit its expression. The promoter sequence of the human TLR2 gene was searched in NCBI database (Supplementary Table S8), indeed, the total 19 putative sites that binding transcription factor Snai3 in the promoter region of TLR2 gene were predicted with relative profile score threshold 80% in JASPAR CORE database (Supplementary Table S9). Moreover, Snai3 treatment resulted in a significant downregulation of TLR2 expression in CMs (Figures 6F,G). These results suggested that jaranol inhibited TAC-induced TLR2 expression was potentially associated with promoting Snai3 expression in mouse heart.

### Identification of NF-κB as a direct jaranol-binding protein

**FIGURE 7:**
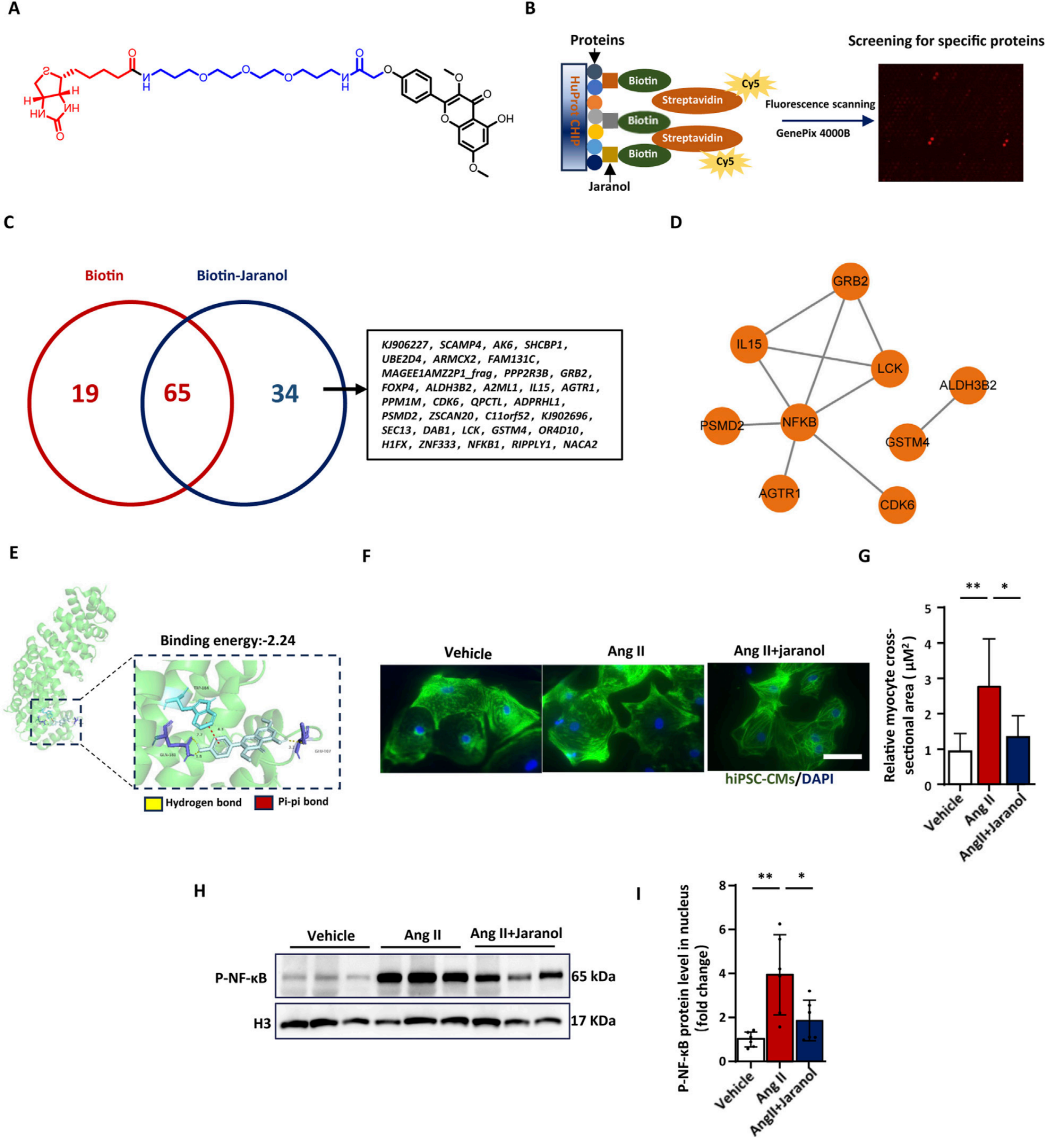
Identification of NF-κB as a direct jaranol-binding protein. **(A)** Biotinylated jaranol; **(B)** protein chip-based proteome microarray assays; **(C)** Venn diagram showing 34 proteins that specifically interact with jaranol; **(D)** protein‒protein interactions revealed by screening 34 proteins; **(E)** the interaction between jaranol and NF-κB was validated by molecular docking; **(F)** immunofluorescence staining of human-induced pluripotent stem cell-derived cardiomyocytes (hiPSC-CMs) treated with or without jaranol during Ang II (angiotensin II)-induced hypertrophy. Scale bar = 50 µm. **(G)** Quantification of the size of hiPSC-CMs treated with or without jaranol during Ang II-induced hypertrophy (n = 6 per group). **(H,I)** Immunoblots and densitometric quantification showing the expression level of P-NF-κB in the nuclei of hiPSC-CMs, with H3 as a control (n = 6 per group). The data are presented as means±SDs; one-way ANOVA with Tukey’s post hoc test; ^*^ *P* < 0.05, ^**^ *P* < 0.01, and ^***^ *P* < 0.001. CMs: cardiomyocytes.

To identify the molecular mechanism by which jaranol prevents TAC-induced cardiac remodelling, we screened for potential jaranol-binding proteins. First, biotinylated jaranol was synthesized (Figure 7A). The mass spectrometry (MS) and nuclear magnetic resonance (NMR) spectra of biotinylated jaranol are shown in Supplementary Figures S11-S12. The biological activity of biotinylated jaranol was evaluated in H9c2 cells, the results of which are shown in Supplementary Figure S13. Following incubation with biotinylated jaranol or free biotin, binding was assessed using Cy5-streptavidin solution (Figure 7B; Supplementary Figure S14). Thirty-four proteins were identified as being able to interact directly with jaranol (Figure 7C; Supplementary Table S10). Cluster analysis was performed using the STRING software and revealed that NF-κB was a hub gene among those proteins (Figure 7D). To validate the interaction between jaranol and NF-κB, molecular docking was performed with software. The results revealed that the binding energies between jaranol and the different sites of NF-κB were less than 0, which illustrated that jaranol is tightly linked to amino acid residues in NF-κB through hydrogen bonds (Figure 7E). To further clarify the mechanism by which jaranol affects cardiac remodelling, hiPSC-CMs were cultured and stimulated with AngII and jaranol. The results revealed a significant inhibitory effect of jaranol against Ang II-induced hiPSC-CM hypertrophy (Figures 7F,G). Besides, we determined the levels of P-NF-κB protein in the nucleus to validate the interaction between jaranol and NF-κB in hiPSC-CMs, the results showed that P-NF-κB level in the nucleus increased in Ang II-induced hiPSC-CMs, which was significantly blunted in jaranol-treated hiPSC-CMs (Figures 7H,I).

## Discussion

This endeavor was intended to investigate the protective effect and mechanism of jaranol against pathological cardiac remodelling *in vitro* and *in vivo*. We have demonstrated for the first time that jaranol protects against TAC- and AngII-induced mouse and cellular cardiac remodelling model by multi-target inhibition of the TLR2 signalling pathway. On the one hand, jaranol promoted transcription factor Snai3 expression in mouse heart to activity the transcriptional repression of TLR2 under the pathological condition, while TLR2 played a critical regulatory role in the development of pathological cardiac remodelling. On the other hand, proteome microarray assays indicated that jaranol could interact with NF-κB, a key regulatory factor of the TLR signalling pathway involved in pathological cardiac remodelling. These conclusions are summarised in the graphical abstract.

Pathological cardiac remodelling is a major risk factor for chronic heart failure, and the prevention and treatment of cardiac remodelling are considered important therapeutic strategies for heart failure (Wang et al., 2023; Bei et al., 2023). Although several risk factors for cardiac remodelling have been identified, including increased cardiac loading, genetic mutations, and epigenetic modification alterations, there are still no effective therapeutic approaches clinically due to complicated mechanisms and a lack of effective targets (Hou and Kang, 2012). Indeed, intensive lowering of blood pressure has been shown to attenuate pathological cardiac remodelling in patients with hypertension, but a subset of hypertensive patients still exhibit a clinical phenotype of cardiac remodelling, especially elderly individuals (Deng et al., 2023; Kanaoka et al., 2013). These results indicated that blood pressure control alone has limitations for the treatment of cardiac remodelling and prevention of heart failure.

Traditional Chinese medicine, including compounds from herbal medicines and classic traditional formulas, has advantages in treating pathological cardiac remodelling (Wang et al., 2024; Wang et al., 2021). Flavonoids, which are widely distributed in botanical medicine, have been reported to have protective effects against cardiac remodelling through their anti-inflammatory, antioxidative, antiapoptotic, and improved effects on myocardial metabolites (Fan et al., 2022; Suchal et al., 2016; Zhu et al., 2019). Like other flavonoids, jaranol also has various bioactivities, such as anticancer, antimicrobial, and antiviral effects, and inhibits cytokine expression (Villaflores et al., 2010; Arenbaoligao et al., 2023). Although previous network pharmacology methods have successfully revealed that jaranol is considered an active ingredient of *Astragalus membranaceus* and multiple formulas, few systematic studies on the activity of jaranol exist (Fang et al., 2022; Zhang and Huang, 2021). Our study revealed that the content of jaranol was low in *Astragalus membranaceus*, which may be why it was ignored in the identification of active ingredients in *Astragalus membranaceus*; this oversight may have served as a key obstacle to further research. In our study, a jaranol standard with a purity greater than 98% was used to treat pathological cardiac remodelling *in vivo* and *in vitro*, ensuring the accuracy of our experimental results.

TLRs played a critical role in pathogenesis of cardiovascular disease. Based on their subcellular localization, TLR1, 2, 4, 5, 6, and 11 are expressed on the plasma membrane, whereas TLR3, 7, 8, and 9 are found in endosomes (Delneste et al., 2007). Except for TLR3, those TLRs interacted with the adaptor protein via myeloid differentiation factor 88 (Myd88)-dependent and independent pathways to activate numerous transcription factors, and subsequently induce inflammation reaction, which was involved in development of cardiovascular disease under the pathological condition (Kawai and Akira, 2011). Among TLRs, TLR2, 3, 4 and 9 have been widely reported to be associated with the occurrence of various cardiovascular diseases, including heart failure, dilated cardiomyopathy, myocardial infarction (Yu and Feng, 2018). In our study, TLR1, 2, 4 and 6 were enriched in TLRs signaling pathway, but the expression of TLR2 in mouse heart was only significantly differential among groups, which was also validated by Western blot. Previous study showed that members of the Snai family, including Snail (Snai1), Slug (Snai2), and Smuc (Snai3), played an important regulatory role in tumorigenesis and drug resistance (Ogihara et al., 2020). At present, although Snai-related proteins as zinc-finger transcription factors had a critical regulatory role in cell-fate determination, there are few reports on relationship between SNAI family and cardiovascular disease, especially for regulatory effect of SNAIs on TLRs expression (Zhuge et al., 2005). Our results showed that transcription factor Snai3 could potentially inhibit TLR2 expression in mouse heart, which was speculated to be related to the binding of Snai2 with the promoter of TLR gene. Besides, the same transcriptional inhibition effect of Snai3 has also been observed in other reports (Batlle et al., 2000; Cano et al., 2000).

TLR-induced inflammatory signalling is pivotal in pathological cardiac remodelling (Zhang et al., 2023). As a type I integral membrane glycoprotein, TLRs are widely distributed in organs and are important components of innate and adaptive immunity (Duan et al., 2022). In the heart, TLRs are activated by pathogen-associated molecular patterns (PAMPs) and damage-associated molecular patterns (DAMPs) under pathological conditions, such as viral infection (Okun et al., 2014), oxidative stress (Nozaki et al., 2004), pressure overload (Oka et al., 2012), and ischaemia‒reperfusion injury (Gollmann-Tepekoylu et al., 2020). Activated TLRs enable cellular signal transduction via myeloid differentiation factor 88 (MyD88)-dependent, MyD88-independent, and TIR domain-containing adaptor-inducing interferon-β (TRIF)-dependent pathways to promote proinflammatory gene expression, prohypertrophic gene expression, and extracellular matrix component expression (Seya et al., 2022; Singh et al., 2012). MyD88 and TRIF, as TLR adaptors, are key in TLR-mediated cellular signal transduction (Krishnan et al., 2007). Previous studies have shown that the signals of all TLR family members, except for TLR3, are transduced through the MyD88-dependent pathway and that TLR4 signals are also transduced through the TRIF-dependent pathway (Lin et al., 2021; Oyama et al., 2012). Interestingly, although TLRs are expressed in various types of cells, the types of TLR-mediated cellular signal transduction differ across different tissues under the same stimulation. A previous study revealed that TLR3- TRIF signalling was activated in Ang II-induced hypertension, whereas TLR4-TRIF and TLR3-TRIF signalling was activated in Ang II-induced cardiac hypertrophy (Singh et al., 2019). In addition to TLR4 and TLR3, TLR2 is involved in pathological cardiac remodelling, as confirmed by reduced cardiac hypertrophy and fibrosis in TLR2 knockout mice compared with those in wild-type mice, providing an important theoretical basis for the cardiac-specific overexpression of TLR2 in our study (Higashikuni et al., 2013). In this study, RNA sequencing of the mouse heart revealed that jaranol treatment inhibited TAC-induced activation of the TLR signalling pathway. Further analysis revealed that the protective effect of jaranol against cardiac remodelling was related to the inhibition of TAC-induced TLR2 expression in the mouse heart; moreover, cardiac-specific TLR2 overexpression exacerbated the protective effect of jaranol against cardiac remodelling.

NF-κB, an inflammatory transcription factor, is involved in various signalling mechanisms, biological processes, and human diseases (Guo et al., 2024). As an important effector molecule, NF-κB is activated in TLR-mediated cellular signal transduction through its translocation from the cytoplasm to the nucleus to regulate the expression of genes, including inflammatory cytokines and factors related to cardiac remodelling (Kawai and Akira, 2007). Some studies have shown that NF-κB is activated through a MyD88-dependent pathway after pressure overload-induced TLR2 activation under pathological conditions (Higashikuni et al., 2013; Zhang D. et al., 2022). NF-κB binds with DNA within particular promoter regions to regulate the expression of more than 200 genes in the heart, including genes involved in NO production, calcium handling, cardiomyocyte function, cell death, extracellular matrix (ECM) remodelling, cell adhesion, and natriuretic factor (ANP and BNP) expression, which are closely associated with the development of cardiac remodelling (Jones et al., 2003). Based on the crucial biological role of NF-κB in pathological cardiac remodelling, NF-κB has been considered a potential therapeutic target for managing cardiac remodelling. Additionally, some flavonoids from TCM, proteins, and traditional formulas have been reported to have protective effects against cardiac remodelling via inhibiting NF-κB activity (Sen and Roy, 2005; Zhang X. et al., 2022; Wu et al., 2009; Li et al., 2022; Muhammad et al., 2019). This is the first study to demonstrate that jaranol significantly inhibited TLR2 activation-related NF-κB signalling, which was achieved by direct interaction between jaranol and NF-κB.

For cardiac remodelling, another pathological presentation is cardiac fibrosis in addition to cardiac hypertrophy. The main cause of cardiac fibrosis is ECM remodelling, which is based on a phenotypic shift in cardiac fibroblasts from myofibroblasts to fibroblasts under pressure overload or other pathological conditions (Frangogiannis, 2021). A previous study revealed that TGF-β signalling is crucial for activating fibroblasts to induce fibrosis reactions via different pathways (Frangogiannis, 2019; Ko et al., 2022; Khalil et al., 2017). We detected a significant increase in TGF-β expression in mouse hearts 4 weeks after TAC, whereas jaranol treatment attenuated TAC-induced TGF-β expression, which is an important reason why jaranol protects against cardiac fibrosis. Although cardiac-specific TLR2 overexpression can lead to severe cardiac fibrosis, we did not observe a significant increase in TGF-β expression in the mouse heart after cardiac-specific TLR2 overexpression. Therefore, we speculated that TLR2 signalling activated cardiac fibrosis in a TGF-β-independent manner.

There are some limitations in this study. Firstly, based on relationship between the effect of jaranol and TLR2 expression in mouse heart, the protective effect of jaranol was validated via cardiac-specific TLR2 overexpression using AAV-9, however, it still remains unclear whether cardiac-specific TLR2 knockout affect jaranol activity. Secondly, the experimental period was 4 weeks, which is a short-term study. Although the previous studies displayed significant cardiac remodelling phenotype in TAC or AngII-induced mice within 4weeks, long-term effectiveness of jaranol on cardiac remodelling and heart failure should be fully assessed with longer experimental cycles (Ju et al., 2024; Liu et al., 2020). Thirdly, limitation to the generalisability of the study is that it did not consider gender/sex issues. The male mice as subjects with a relatively limited sample size were used in this study, which partly attributed to the protective effect of oestrogen on cardiovascular disease, and sex differences as specific risk factors influencing the prevalence of cardiovascular disease in unique ways (Colafella and Denton, 2018). Therefore, the efficacy and safety of jaranol in different sexes need to be evaluated in future studies. Finally, although jaranol displayed a potential bioactivity against pathological cardiac remodelling via multi-target inhibition of Snai3/TLR2 and NF-κB signalling pathways in animal experiments, it still has a long way to go to translate these results into clinical applications. Future studies are needed to elucidate its structure-activity relationship, tissue distribution characteristics, pharmacokinetic characteristics, et al.

## Conclusion

Collectively, our findings highlight the protective role of jaranol against cardiac remodelling via multi-target inhibition of Snai3/TLR2 and NF-κB signaling pathways and suggest that jaranol, which targets NF-κB, is a feasible and promising option for the prevention or treatment of cardiac remodelling. In the future, we will focus on constitutive relationship-based structural modifications to improve the activity and physical properties of jaranol to provide a basis for further clinical translation.

## Data Availability

The datasets presented in this study can be found in online repositories. The names of the repository/repositories and accession number(s) can be found below: https://www.ncbi.nlm.nih.gov/, PRJNA1150657.

## Abbreviations

WGCNA, weighted gene co-expression network analysis; Ang II, angiotensin II; TLR, Toll-like receptor; TAC, transverse aortic constriction; ACEIs/ARBs, angiotensin-converting enzyme/receptor inhibitors; CCBs, channel blockers; TCMSP, Traditional Chinese Medicine Systems Pharmacology; CMs, cardiomyocytes; HPLC, high-pressure liquid chromatography; CFs, cardiac fibroblasts; hIPSC-CMs, Human induced pluripotent stem cell-derived cardiomyocytes; AAV-9, Adeno-associated virus-9; GFP, green fluorescent protein; Anp, atrial natriuretic peptide; Bnp, brain natriuretic peptide; GAPDH, glyceraldehyde-3-phosphate dehydrogenase; LVPWd, left ventricle posterior wall thickness in diastole; EF, left ventricular ejection fraction; FS, left ventricular shortening fraction.

## References

[B1] Arenbaoligao A. Guo X. Xiong J. Zhang S. Yang Y. Chen D. (2023). Kumatakenin inhibited iron-ferroptosis in epithelial cells from colitis mice by regulating the Eno3-IRP1-axis. Front. Pharmacol. 14, 1127931. 10.3389/fphar.2023.1127931 37006994 PMC10063804

[B2] Batlle E. Sancho E. Franci C. Dominguez d. Monfar m. Baulida J. (2000). The transcription factor snail is a repressor of E-cadherin gene expression in epithelial tumour cells. Nat. Cell Biol. 2, 84–89. 10.1038/35000034 10655587

[B3] Bei Y. Zhu Y. Wei M. Yin M. Li L. Chen C. (2023). HIPK1 inhibition protects against pathological cardiac hypertrophy by inhibiting the CREB-C/EBPβ axis. Adv. Sci. (Weinh) 10, e2300585. 10.1002/advs.202300585 37098980 PMC10288234

[B4] Cano A. Perez-Moreno M. A. Rodrigo I. Locascio A. Blanco M. J. Del Barrio M. G. (2000). The transcription factor snail controls epithelial-mesenchymal transitions by repressing E-cadherin expression. Nat. Cell Biol. 2, 76–83. 10.1038/35000025 10655586

[B5] Chen C. Y. (2011). TCM database@Taiwan: the world's largest traditional Chinese medicine database for drug screening *in silico* . PLoS One 6, e15939. 10.1371/journal.pone.0015939 21253603 PMC3017089

[B6] Chen Q. Q. Ma G. Liu J. F. Cai Y. Y. Zhang J. Y. Wei T. T. (2021). Neuraminidase 1 is a driver of experimental cardiac hypertrophy. Eur. Heart J. 42, 3770–3782. 10.1093/eurheartj/ehab347 34179969

[B7] Colafella K. M. M. Denton K. M. (2018). Sex-specific differences in hypertension and associated cardiovascular disease. Nat. Rev. Nephrol. 14, 185–201. 10.1038/nrneph.2017.189 29380817

[B8] Daina A. Michielin O. Zoete V. (2019). SwissTargetPrediction: updated data and new features for efficient prediction of protein targets of small molecules. Nucleic Acids Res. 47, W357–W364. 10.1093/nar/gkz382 31106366 PMC6602486

[B9] Dao T. B. Nguyen T. M. Nguyen V. Q. Tran T. M. Tran N. M. Nguyen C. H. (2021). Flavones from Combretum quadrangulare growing in Vietnam and their alpha-glucosidase inhibitory activity. Molecules 26, 2531. 10.3390/molecules26092531 33926133 PMC8123651

[B10] Delneste Y. Beauvillain C. Jeannin P. (2007). Innate immunity: structure and function of TLRs. Med. Sci. Paris. 23, 67–73. 10.1051/medsci/200723167 17212934

[B11] Deng Y. Liu W. Yang X. Guo Z. Zhang J. Huang R. (2023). Intensive blood pressure lowering improves left ventricular hypertrophy in older patients with hypertension: the STEP trial. Hypertension 80, 1834–1842. 10.1161/HYPERTENSIONAHA.122.20732 37259845 PMC10424814

[B12] DennisG.JR. ShermanB. T. HosackD. A. YangJ. GaoW. LaneH. C. (2003). DAVID: database for annotation, visualization, and integrated discovery. Genome Biol. 4, P3. 10.1186/gb-2003-4-5-p3 12734009

[B13] Duan T. Du Y. Xing C. Wang H. Y. Wang R. F. (2022). Toll-like receptor signaling and its role in cell-mediated immunity. Front. Immunol. 13, 812774. 10.3389/fimmu.2022.812774 35309296 PMC8927970

[B14] Fan S. Hu Y. You Y. Xue W. Chai R. Zhang X. (2022). Role of resveratrol in inhibiting pathological cardiac remodeling. Front. Pharmacol. 13, 924473. 10.3389/fphar.2022.924473 36120366 PMC9475218

[B15] Fang J. Wang C. Zheng J. Liu Y. (2022). Network pharmacology study of yishen capsules in the treatment of diabetic nephropathy. PLoS One 17, e0273498. 10.1371/journal.pone.0273498 36094934 PMC9467320

[B16] Frangogiannis N. G. (2019). Cardiac fibrosis: cell biological mechanisms, molecular pathways and therapeutic opportunities. Mol. Asp. Med. 65, 70–99. 10.1016/j.mam.2018.07.001 30056242

[B17] Frangogiannis N. G. (2021). Cardiac fibrosis. Cardiovasc Res. 117, 1450–1488. 10.1093/cvr/cvaa324 33135058 PMC8152700

[B18] Gao L. Yao R. Liu Y. Wang Z. Huang Z. Du B. (2017). Isorhamnetin protects against cardiac hypertrophy through blocking PI3K-AKT pathway. Mol. Cell Biochem. 429, 167–177. 10.1007/s11010-017-2944-x 28176246

[B19] Gollmann-Tepekoylu C. Graber M. Polzl L. Nagele F. Moling R. Esser H. (2020). Toll-like receptor 3 mediates ischaemia/reperfusion injury after cardiac transplantation. Eur. J. Cardiothorac. Surg. 57, 826–835. 10.1093/ejcts/ezz383 32040169

[B20] Guo Z. Lou Y. Kong M. Luo Q. Liu Z. Wu J. (2019). A systematic review of phytochemistry, pharmacology and pharmacokinetics on astragali radix: implications for astragali radix as a personalized medicine. Int. J. Mol. Sci. 20, 1463. 10.3390/ijms20061463 30909474 PMC6470777

[B21] Guo Q. Jin Y. Chen X. Ye X. Shen X. Lin M. (2024). NF-κB in biology and targeted therapy: new insights and translational implications. Signal Transduct. Target Ther. 9, 53. 10.1038/s41392-024-01757-9 38433280 PMC10910037

[B22] Higashikuni Y. Tanaka K. Kato M. Nureki O. Hirata Y. Nagai R. (2013). Toll-like receptor-2 mediates adaptive cardiac hypertrophy in response to pressure overload through interleukin-1β upregulation *via* nuclear factor κB activation. J. Am. Heart Assoc. 2, e000267. 10.1161/JAHA.113.000267 24249711 PMC3886766

[B23] Hou J. Kang Y. J. (2012). Regression of pathological cardiac hypertrophy: signaling pathways and therapeutic targets. Pharmacol. Ther. 135, 337–354. 10.1016/j.pharmthera.2012.06.006 22750195 PMC3458709

[B24] Jones W. K. Brown M. Ren X. He S. Mcguinness M. (2003). NF-kappaB as an integrator of diverse signaling pathways: the heart of myocardial signaling? Cardiovasc Toxicol. 3, 229–254. 10.1385/ct:3:3:229 14555789

[B25] Ju J. Wang K. Liu F. Liu C. Y. Wang Y. H. Wang S. C. (2024). Crotonylation of NAE1 modulates cardiac hypertrophy *via* gelsolin neddylation. Circ. Res. 135, 806–821. 10.1161/CIRCRESAHA.124.324733 39229723

[B26] Kanaoka T. Tamura K. Wakui H. Ohsawa M. Azushima K. Uneda K. (2013). L/N-type calcium channel blocker cilnidipine added to renin-angiotensin inhibition improves ambulatory blood pressure profile and suppresses cardiac hypertrophy in hypertension with chronic kidney disease. Int. J. Mol. Sci. 14, 16866–16881. 10.3390/ijms140816866 23959116 PMC3759940

[B27] Kawai T. Akira S. (2007). Signaling to NF-kappaB by toll-like receptors. Trends Mol. Med. 13, 460–469. 10.1016/j.molmed.2007.09.002 18029230

[B28] Kawai T. Akira S. (2011). Toll-like receptors and their crosstalk with other innate receptors in infection and immunity. Immunity 34, 637–650. 10.1016/j.immuni.2011.05.006 21616434

[B29] Khalil H. Kanisicak O. Prasad V. Correll R. N. Fu X. Schips T. (2017). Fibroblast-specific TGF-beta-Smad2/3 signaling underlies cardiac fibrosis. J. Clin. Invest 127, 3770–3783. 10.1172/JCI94753 28891814 PMC5617658

[B30] Ko T. Nomura S. Yamada S. Fujita K. Fujita T. Satoh M. (2022). Cardiac fibroblasts regulate the development of heart failure *via* Htra3-TGF-beta-IGFBP7 axis. Nat. Commun. 13, 3275. 10.1038/s41467-022-30630-y 35672400 PMC9174232

[B31] Krishnan J. Selvarajoo K. Tsuchiya M. Lee G. Choi S. (2007). Toll-like receptor signal transduction. Exp. Mol. Med. 39, 421–438. 10.1038/emm.2007.47 17934330

[B32] Leal C. M. Leitao S. G. Sausset R. Mendonca S. C. Nascimento P. H. A. De araujo R. C. C. F. (2021). Flavonoids from Siparuna cristata as potential inhibitors of SARS-CoV-2 replication. Rev. Bras. Farmacogn. 31, 658–666. 10.1007/s43450-021-00162-5 34305198 PMC8294293

[B33] Li D. Guo Y. Y. Cen X. F. Qiu H. L. Chen S. Zeng X. F. (2022). Lupeol protects against cardiac hypertrophy *via* TLR4-PI3K-Akt-NF-κB pathways. Acta Pharmacol. Sin. 43, 1989–2002. 10.1038/s41401-021-00820-3 34916609 PMC9343642

[B34] Lin C. Wang H. Zhang M. Mustafa S. Wang Y. Li H. (2021). TLR4 biased small molecule modulators. Pharmacol. Ther. 228, 107918. 10.1016/j.pharmthera.2021.107918 34171331

[B35] Lipinski C. A. (2004). Lead- and drug-like compounds: the rule-of-five revolution. Drug Discov. Today Technol. 1, 337–341. 10.1016/j.ddtec.2004.11.007 24981612

[B36] Liu Y. Gao L. Guo S. Liu Y. Zhao X. Li R. (2017). Kaempferol alleviates angiotensin II-Induced cardiac dysfunction and interstitial fibrosis in mice. Cell Physiol. Biochem. 43, 2253–2263. 10.1159/000484304 29073623

[B37] Liu Y. Lu H. Zhang C. Hu J. Xu D. (2019). Recent advances in understanding the roles of T cells in pressure overload-induced cardiac hypertrophy and remodeling. J. Mol. Cell Cardiol. 129, 293–302. 10.1016/j.yjmcc.2019.01.005 30641087

[B38] Liu T. Wen H. Li H. Xu H. Xiao N. Liu R. (2020). Oleic acid attenuates ang II (angiotensin II)-induced cardiac remodeling by inhibiting FGF23 (fibroblast growth factor 23) expression in mice. Hypertension 75, 680–692. 10.1161/HYPERTENSIONAHA.119.14167 31928110

[B39] Liu T. Zhang Y. Liu J. Peng J. Jia X. Xiao Y. (2022). Evaluation of the acute and sub-acute oral toxicity of jaranol in kunming mice. Front. Pharmacol. 13, 903232. 10.3389/fphar.2022.903232 35847023 PMC9280858

[B40] Liu Y. X. Song X. M. Dan L. W. Tang J. M. Jiang Y. Deng C. (2024). Astragali radix: comprehensive review of its botany, phytochemistry, pharmacology and clinical application. Arch. Pharm. Res. 47, 165–218. 10.1007/s12272-024-01489-y 38493280

[B41] Muhammad T. Ikram M. Ullah R. Rehman S. U. Kim M. O. (2019). Hesperetin, a citrus flavonoid, attenuates LPS-induced neuroinflammation, apoptosis and memory impairments by modulating TLR4/NF-kappaB signaling. Nutrients 11. 10.3390/nu11030648 30884890 PMC6471991

[B42] Nakamura M. Sadoshima J. (2018). Mechanisms of physiological and pathological cardiac hypertrophy. Nat. Rev. Cardiol. 15, 387–407. 10.1038/s41569-018-0007-y 29674714

[B43] Nozaki N. Shishido T. Takeishi Y. Kubota I. (2004). Modulation of doxorubicin-induced cardiac dysfunction in toll-like receptor-2-knockout mice. Circulation 110, 2869–2874. 10.1161/01.CIR.0000146889.46519.27 15505089

[B44] Ogihara T. Mizoi K. Kamioka H. Yano K. (2020). Physiological roles of ERM proteins and transcriptional regulators in supporting membrane expression of efflux transporters as factors of drug resistance in cancer. Cancers (Basel) 12, 3352. 10.3390/cancers12113352 33198344 PMC7696277

[B45] Oka T. Hikoso S. Yamaguchi O. Taneike M. Takeda T. Tamai T. (2012). Mitochondrial DNA that escapes from autophagy causes inflammation and heart failure. Nature 485, 251–255. 10.1038/nature10992 22535248 PMC3378041

[B46] Okun E. Griffioen K. J. Rothman S. Wan R. Cong W. N. De cabo R. (2014). Toll-like receptors 2 and 4 modulate autonomic control of heart rate and energy metabolism. Brain Behav. Immun. 36, 90–100. 10.1016/j.bbi.2013.10.013 24145051 PMC3947180

[B47] Oyama J. Maeda T. Sasaki M. Higuchi Y. Node K. Makino N. (2012). Repetitive hyperthermia attenuates progression of left ventricular hypertrophy and increases telomerase activity in hypertensive rats. Am. J. Physiol. Heart Circ. Physiol. 302, H2092–H2101. 10.1152/ajpheart.00225.2011 22427516

[B48] Qian L. Xu H. Yuan R. Yun W. Ma Y. (2024). Formononetin ameliorates isoproterenol induced cardiac fibrosis through improving mitochondrial dysfunction. Biomed. Pharmacother. 170, 116000. 10.1016/j.biopha.2023.116000 38070245

[B49] Quek A. Mohd zaini H. Kassim N. K. Sulaiman F. Rukayadi Y. Ismail A. (2021). Oxygen radical antioxidant capacity (ORAC) and antibacterial properties of Melicope glabra bark extracts and isolated compounds. PLoS One 16, e0251534. 10.1371/journal.pone.0251534 33970960 PMC8109830

[B50] Ritterhoff J. Tian R. (2023). Metabolic mechanisms in physiological and pathological cardiac hypertrophy: new paradigms and challenges. Nat. Rev. Cardiol. 20, 812–829. 10.1038/s41569-023-00887-x 37237146

[B51] Schnelle M. Sawyer I. Anilkumar N. Mohamed B. A. Richards D. A. Toischer K. (2021). NADPH oxidase-4 promotes eccentric cardiac hypertrophy in response to volume overload. Cardiovasc Res. 117, 178–187. 10.1093/cvr/cvz331 31821410 PMC7797217

[B52] Sen C. K. Roy S. (2005). Relief from a heavy heart: redox-sensitive NF-kappaB as a therapeutic target in managing cardiac hypertrophy. Am. J. Physiol. Heart Circ. Physiol. 289, H17–H19. 10.1152/ajpheart.00250.2005 15961373

[B53] Seya T. Tatematsu M. Matsumoto M. (2022). Toward establishing an ideal adjuvant for non-inflammatory immune enhancement. Cells 11, 4006. 10.3390/cells11244006 36552770 PMC9777512

[B54] Singh M. V. Swaminathan P. D. Luczak E. D. Kutschke W. Weiss R. M. Anderson M. E. (2012). MyD88 mediated inflammatory signaling leads to CaMKII oxidation, cardiac hypertrophy and death after myocardial infarction. J. Mol. Cell Cardiol. 52, 1135–1144. 10.1016/j.yjmcc.2012.01.021 22326848 PMC3327770

[B55] Singh M. V. Cicha M. Z. Nunez S. Meyerholz D. K. Chapleau M. W. Abboud F. M. (2019). Angiotensin II-induced hypertension and cardiac hypertrophy are differentially mediated by TLR3-and TLR4-dependent pathways. Am. J. Physiol. Heart Circ. Physiol. 316, H1027–H1038. 10.1152/ajpheart.00697.2018 30793936 PMC6580398

[B56] Suchal K. Malik S. Gamad N. Malhotra R. K. Goyal S. N. Bhatia J. (2016). Kampeferol protects against oxidative stress and apoptotic damage in experimental model of isoproterenol-induced cardiac toxicity in rats. Phytomedicine 23, 1401–1408. 10.1016/j.phymed.2016.07.015 27765360

[B57] Takemoto Y. Yoshiyama M. Takeuchi K. Omura T. Komatsu R. Izumi Y. (1999). Increased JNK, AP-1 and NF-kappa B DNA binding activities in isoproterenol-induced cardiac remodeling. J. Mol. Cell Cardiol. 31, 2017–2030. 10.1006/jmcc.1999.1033 10591028

[B58] Tavakoli R. Nemska S. Jamshidi P. Gassmann M. Frossard N. (2017). Technique of minimally invasive transverse aortic constriction in mice for induction of left ventricular hypertrophy. J. Vis. Exp., 56231. 10.3791/56231 28994784 PMC5752328

[B59] Villaflores O. B. Macabeo A. P. Gehle D. Krohn K. Franzblau S. G. Aguinaldo A. M. (2010). Phytoconstituents from Alpinia purpurata and their *in vitro* inhibitory activity against *Mycobacterium tuberculosis* . Pharmacogn. Mag. 6, 339–344. 10.4103/0973-1296.71785 21120040 PMC2992151

[B60] Wang L. Li Y. L. Zhang C. C. Cui W. Wang X. Xia Y. (2014). Inhibition of toll-like receptor 2 reduces cardiac fibrosis by attenuating macrophage-mediated inflammation. Cardiovasc Res. 101, 383–392. 10.1093/cvr/cvt258 24259498

[B61] Wang L. Zhang Y. L. Lin Q. Y. Liu Y. Guan X. M. Ma X. L. (2018). CXCL1-CXCR2 axis mediates angiotensin II-induced cardiac hypertrophy and remodelling through regulation of monocyte infiltration. Eur. Heart J. 39, 1818–1831. 10.1093/eurheartj/ehy085 29514257

[B62] Wang Y. Wu J. Wang D. Yang R. Liu Q. (2021). Traditional Chinese medicine targeting heat shock proteins as therapeutic strategy for heart failure. Front. Pharmacol. 12, 814243. 10.3389/fphar.2021.814243 35115946 PMC8804377

[B63] Wang M. Han X. Yu T. Wang M. Luo W. Zou C. (2023). OTUD1 promotes pathological cardiac remodeling and heart failure by targeting STAT3 in cardiomyocytes. Theranostics 13, 2263–2280. 10.7150/thno.83340 37153745 PMC10157730

[B64] Wang J. Zou J. Shi Y. Zeng N. Guo D. Wang H. (2024). Traditional Chinese medicine and mitophagy: a novel approach for cardiovascular disease management. Phytomedicine 128, 155472. 10.1016/j.phymed.2024.155472 38461630

[B65] Woo J. H. Ahn J. H. jang D. S. Lee K. T. Choi J. H. (2017). Effect of kumatakenin isolated from cloves on the apoptosis of cancer cells and the alternative activation of tumor-associated macrophages. J. Agric. Food Chem. 65, 7893–7899. 10.1021/acs.jafc.7b01543 28763204

[B66] Wu S. Yin R. Ernest R. Li Y. Zhelyabovska O. Luo J. (2009). Liver X receptors are negative regulators of cardiac hypertrophy *via* suppressing NF-kappaB signalling. Cardiovasc Res. 84, 119–126. 10.1093/cvr/cvp180 19487338 PMC2741346

[B67] Xue R. Fang Z. Zhang M. Yi Z. Wen C. Shi T. (2013). TCMID: traditional Chinese medicine integrative database for herb molecular mechanism analysis. Nucleic Acids Res. 41, D1089–D1095. 10.1093/nar/gks1100 23203875 PMC3531123

[B68] Yu L. Feng Z. (2018). The role of toll-like receptor signaling in the progression of heart failure. Mediat. Inflamm. 2018, 9874109. 10.1155/2018/9874109 29576748 PMC5822798

[B69] Zhang Q. Huang X. (2021). The modulatory properties of Astragalus membranaceus treatment on endometrial cancer: an integrated pharmacological method. PeerJ 9, e11995. 10.7717/peerj.11995 34513331 PMC8395571

[B70] Zhang X. Wang L. Mu H. Wang D. Yu Y. (2019). Synergistic antibacterial effects of Buddleja albiflora metabolites with antibiotics against Listeria monocytogenes. Lett. Appl. Microbiol. 68, 38–47. 10.1111/lam.13084 30298931

[B71] Zhang D. Li Y. Wang W. Lang X. Zhang Y. Zhao Q. (2022a). NOX1 promotes myocardial fibrosis and cardiac dysfunction *via* activating the TLR2/NF-κB pathway in diabetic cardiomyopathy. Front. Pharmacol. 13, 928762. 10.3389/fphar.2022.928762 36225554 PMC9549956

[B72] Zhang X. Qu H. Yang T. Liu Q. Zhao D. Liu W. (2022b). LuQi formula ameliorates myocardial fibrosis by suppressing TLR4/MyD88/NF-*κ*B pathway and NLRP3 inflammasome activation in mice with myocardial infarction. Evid. Based Complement. Altern. Med. 2022, 5867987. 10.1155/2022/5867987 35310035 PMC8933100

[B73] Zhang Y. Wu J. Dong E. Wang Z. Xiao H. (2023). Toll-like receptors in cardiac hypertrophy. Front. Cardiovasc Med. 10, 1143583. 10.3389/fcvm.2023.1143583 37113698 PMC10126280

[B74] Zhou Q. Meng D. Li F. Zhang X. Liu L. Zhu Y. (2022). Inhibition of HIPK2 protects stress-induced pathological cardiac remodeling. EBioMedicine 85, 104274. 10.1016/j.ebiom.2022.104274 36182775 PMC9526139

[B75] Zhu Z. Y. Wang F. Jia C. H. Xie M. L. (2019). Apigenin-induced HIF-1α inhibitory effect improves abnormal glucolipid metabolism in AngⅡ/hypoxia-stimulated or HIF-1α-overexpressed H9c2 cells. Phytomedicine 62, 152713. 10.1016/j.phymed.2018.10.010 31078968

[B76] Zhuang L. Jia K. Chen C. Li Z. Zhao J. Hu J. (2022). DYRK1B-STAT3 drives cardiac hypertrophy and heart failure by impairing mitochondrial bioenergetics. Circulation 145, 829–846. 10.1161/CIRCULATIONAHA.121.055727 35235343

[B77] Zhuge X. Kataoka H. Tanaka M. Murayama T. Kawamoto T. Sano H. (2005). Expression of the novel Snai-related zinc-finger transcription factor gene smuc during mouse development. Int. J. Mol. Med. 15, 945–948. 10.3892/ijmm.15.6.945 15870897

