# Supplementary material for: Jaranol: a compound with therapeutic activity against pathological cardiac remodelling via multi-target inhibition of the Snai3/TLR2 and NF-κB signaling pathways
Source: Front Pharmacol. 2026 Jan 2;16:1723889. doi: 10.3389/fphar.2025.1723889 (PMC12807897; doi:10.3389/fphar.2025.1723889)
Supplement: Supplementary file 1 [file Supplementaryfile1.docx]

**SUPPLEMENTAL MATERIAL**

**Jaranol: a compound with therapeutic activity against pathological cardiac remodelling via multi-target inhibition of the Snai3/TLR2 and NF-κB signaling pathways**

Yao Zhang^1,2,#^, Nan Li^1,2#^, Shiqi Chu^1,2#^, Heqing Fu^1,2^, Xiaowen Yang^1^, Huimin Xia^3^, Yunfeng Xiao^4^, Zhibin Xiao^5^, Jing Liu^1,2^, Yu Dong^3,6*^, Tianlong Liu^1,2*^

1. Department of Pharmacy, Affiliated Hospital of Inner Mongolia Medical University, Hohhot 010059, China.

2. Key Laboratory of Clinical and Basic Research on Cardiovascular Diseases, Basic Research Team of Cardiovascular Diseases, Affiliated Hospital of Inner Mongolia Medical University, Hohhot 010059, China.

3. Department of Natural Medicinal Chemistry, College of Pharmacy, Inner Mongolia Medical University, Hohhot 010110, China

4. Center for New Drug Safety Evaluation and Research, Inner Mongolia Medical University, Hohhot 010110, China.

5. Department of Clinical Pharmacy, College of Pharmacy, Inner Mongolia Medical University, Hohhot 010110, China.

6. Engineering Technology Research Center of Pharmacodynamic Substance and Quality Control of Mongolian Medicine in Inner Mongolia, Hohhot 010110, China

^#^ These authors contributed equally to this work.

^*^**Correspondence to:**

**Yu Dong, PhD, 3)** Department of Natural Medicinal Chemistry, College of Pharmacy, Inner Mongolia Medical University, Chilechuan Dairy Development Zone, Hohhot, Inner Mongolia, 010110, China. **6)** Engineering Technology Research Center of Pharmacodynamic Substance and Quality Control of Mongolian Medicine in Inner Mongolia, Inner Mongolia Medical University, Chilechuan Dairy Development Zone, Hohhot, Inner Mongolia, 010110, China.

E-mail: 20010040@immu.edu.cn

Phone: Tel: +86-13644887629

**Tianlong Liu, PhD, 1)** Department of Pharmacy, Affiliated Hospital of Inner Mongolia Medical University, No. 1, Tongdao North Street, Huimin District, Hohhot 010059, China; **2)** Key Laboratory of Clinical and Basic Research on Cardiovascular Diseases, Basic Research Team of Cardiovascular Diseases, Inner Mongolia Medical University, No. 1, Tongdao North Street, Huimin District, Hohhot 010059, China.

E-mail: tianlongliu1984@immu.edu.cn

Phone: Tel: +86-0471-3451657

**Table S1** Primer sequences for the real-time qPCR assay

| **Gene** | **Species** | | **Premier** |
| --- | --- | --- | --- |
| Anp | Mouse | Forward primer | 5'-CggAgCCTACgAAgATCCAg-3' |
|  |  | Reverse primer | 5'-AAgCTgTTgCAgCCTAgTCC-3' |
| Bnp | Mouse | Forward primer | 5'-gAggTCACTCCTATCCTCTgg-3' |
|  |  | Reverse primer | 5'-gCCATTTCCTCCgACTTTTCTC-3' |
| Gapdh | Mouse | Forward primer | 5'-AggTCggTgTgAACggATTTg-3' |
|  |  | Reverse primer | 5'-TgTAgACCATgTAgTTgAggTCA-3' |
| Col1α1 | Mouse | Forward primer | 5'-TTCTTCTggCAAAgACggAC-3' |
|  |  | Reverse primer | 5'-CCATCggTCATgCTCTCTCC-3' |
| Col3α1 | Mouse | Forward primer | 5'-CTgTAACATggAAACTggggAAA-3' |
|  |  | Reverse primer | 5'-CCATAgCTgAACTgAAAACCACC-3' |
| α-SMA | Mouse | Forward primer | 5'-gTCCCAgACATCAgggAgTAA-3' |
|  |  | Reverse primer | 5'-TCggATACTTCAgCgTCAggA-3' |

**Table S2** The baseline characteristics of patients with heart failure and controls

| **Characteristics** | **HF** | **controls** | **P value** |
| --- | --- | --- | --- |
| N | 20 | 20 | - |
| Men, n,% | 10,50% | 10,50% | - |
| Age (years) | 58.18±13.62 | 58.89±11.27 | 0.85 |
| Body weight (Kg) | 68.81±13.39 | 67.32±15.36 | 0.74 |
| BMI (Kg/m2) | 24.98±4.92 | 21±3.25 | 0.004 |
| SBP(mm Hg) | 125.76±19.21 | 106.32±22.05 | 0.005 |
| DBP (mm Hg) | 79.65±14.22 | 86.25±20.36 | 0.24 |
| Urea (μmol/L) | 8.87±3.42 | 3.17±1.16 | <0.0001 |
| Cre (μmol/L) | 76.18±32.21 | 51.01±16.23 | 0.003 |
| UA (μmol/L) | 430.53±174.84 | 183.32±86.23 | <0.0001 |
| NTproBNP (pg/ml) | 4932.78±4867.44 | 285.12±56.01 | <0.0001 |
| LADs（mm) | 44.88±10.25 | 35.72±3.46 | 0.0005 |
| AODd（mm) | 30.92±3.78 | 29.06±6.06 | 0.25 |
| RVDd (mm) | 38.53±7.19 | 22.67±1.08 | <0.0001 |
| MPA (mm） | 22.92±3.82 | 21.28±1.93 | 0.094 |
| RADs（mm) | 39.88±8.55 | 34.83±6.77 | 0.045 |
| IVSd（mm） | 8.09±0.7 | 8.61±1.72 | 0.22 |
| LVPWd（mm） | 8.09±0.7 | 8.56±1.2 | 0.14 |
| LVDd(mm) | 58.18±10.94 | 49.44±2.81 | 0.001 |
| LVDs (mm） | 45.4±9.18 | 28.33±2.61 | <0.0001 |
| LVM, g | 185.66±64.02 | 147.05±22.38 | 0.015 |
| LVMI(g/m2) | 107.54±39.51 | 85.22±14.76 | 0.023 |
| EF（%） | 41.71±9.52 | 73.17±4.27 | <0.0001 |
| FS（%） | 20.67±5.87 | 42.61±3.82 | <0.0001 |

Note: HF, heart failure; BMI, Body Mass Index, BMI = body weight[kg]÷(Height[m²]); SBP, systolic blood pressure; DBP, diastolic blood pressure; Cre, creatinine; UA, uric acid; NTproBNP, N-terminal pro b-type natriuretic peptide; LADs, left atrial diameters; AODd, aorta diameter; RVDd, right ventricular diastolic diameter; MPA, main pulmonary artery diameter; RADs, right atrial diastolic diameter; IVSd, interventricular septal thickness; LVPWd, left ventricle posterior wall thickness in diastole; LVDd, left ventricular end-diastolic diameter; LVDs, left ventricular diastolic dimensions; LVM, left ventricular mass, LVM (g) = 0.8{1.04[([LVEDD + IVSd + PWd]3 − LVEDD3)]} + 0.6; LVMI, left ventricular mass index, BSA (m²) = 0.20247 x height(m)^0.725 x body weight(kg)^0.425, LVMI=LVM/BSA; EF, left ventricular ejection fraction; FS, left ventricular shortening fraction. Data are expressed as mean ± standard deviation or as number (percentage), χ^2^ test, and student’s *t*-test were used.

**Table S3** The ingredients of *Astragalus membranaceus*

| **NO** | **Ingredients** | **SMILES** | **Molecular weight** |
| --- | --- | --- | --- |
| 1 | vanillic acid | COC1=C(C=CC(=C1)C(=O)O)O | 168.16 |
| 2 | Linoleic Acid | CCCCCC=CCC=CCCCCCCCC(=O)O | 280.5 |
| 3 | Mairin | CC(=C)C1CCC2(C1C3CCC4C5(CCC(C(C5CCC4(C3(CC2)C)C)(C)C)O)C)C(=O)O | 456.78 |
| 4 | Heriguard | C1C(C(C(CC1(C(=O)O)O)OC(=O)C=CC2=CC(=C(C=C2)O)O)O)O | 354.34 |
| 5 | Jaranol | COC1=CC(=C2C(=C1)OC(=C(C2=O)OC)C3=CC=C(C=C3)O)O | 314.31 |
| 6 | Rhamnocitrin | COC1=CC(=C2C(=C1)OC(=C(C2=O)O)C3=CC=C(C=C3)O)O | 300.28 |
| 7 | alexandrin | CCC(CCC(C)C1CCC2C1(CCC3C2CC=C4C3(CCC(C4)OC5C(C(C(C(O5)CO)O)O)O)C)C)C(C)C | 576.95 |
| 8 | hederagenin | CC1(CCC2(CCC3(C(=CCC4C3(CCC5C4(CCC(C5(C)CO)O)C)C)C2C1)C)C(=O)O)C | 414.79 |
| 9 | (3S,8S,9S,10R,13R,14S,17R)-10,13-dimethyl-17-[(2R,5S)-5-propan-2-yloctan-2-yl]-2,3,4,7,8,9,11,12,14,15,16,17-dodecahydro-1H-cyclopenta[a]phenanthren-3-ol | CCCC(CCC(C)C1CCC2C1(CCC3C2CC=C4C3(CCC(C4)O)C)C)C(C)C | 428.82 |
| 10 | isorhamnetin | COC1=C(C=CC(=C1)C2=C(C(=O)C3=C(C=C(C=C3O2)O)O)O)O | 316.28 |
| 11 | lupeol | CC(=C)C1CCC2(C1C3CCC4C5(CCC(C(C5CCC4(C3(CC2)C)C)(C)C)O)C)C | 426.8 |
| 12 | 3,9-di-O-methylnissolin | COC1=CC2=C(C=C1)C3C(CO2)C4=C(O3)C(=C(C=C4)OC)OC | 314.36 |
| 13 | 3-Hydroxy-2-picoline | CC1=C(C=CC=N1)O | 109.14 |
| 14 | (2S)-4-methoxy-7-methyl-2-[2-[(2S,3R,4S,5S,6R)-3,4,5-trihydroxy-6-(hydroxymethyl)oxan-2-yl]oxypropan-2-yl]-2,3-dihydrofuro[3,2-g]chromen-5-one | CC1=CC(=O)C2=C(C3=C(C=C2O1)OC(C3)C(C)(C)OC4C(C(C(C(O4)CO)O)O)O)OC | 452.5 |
| 15 | 7,2'-Dihydroxy-3',4'-dimethoxyisoflavone 7-O-glucoside | COC1=C(C(=C(C=C1)C2=COC3=C(C2=O)C=CC(=C3)OC4C(C(C(C(O4)CO)O)O)O)O)OC | 476.47 |
| 16 | 7-hydroxy-3-(2-hydroxy-3,4-dimethoxy-phenyl)chromone | COC1=C(C(=C(C=C1)C2=COC3=C(C2=O)C=CC(=C3)O)O)OC | 314.31 |
| 17 | 7-O-methylisomucronulatol | COC1=CC2=C(CC(CO2)C3=C(C(=C(C=C3)OC)OC)O)C=C1 | 316.38 |
| 18 | 9,10-dimethoxypterocarpan-3-O-beta-D-glucoside | COC1=C(C2=C(C=C1)C3COC4=C(C3O2)C=CC(=C4)OC5C(C(C(C(O5)CO)O)O)O)OC | 462.49 |
| 19 | (6aR,11aR)-9,10-dimethoxy-6a,11a-dihydro-6H-[1]benzofuro[3,2-c]chromen-3-ol | COC1=C(C2=C(C=C1)C3COC4=C(C3O2)C=CC(=C4)O)OC | 300.33 |
| 20 | 13-hydroxy-9,11-octadecadienoic acid | CCCCCC(C=CC=CCCCCCCCC(=O)O)O | 296.5 |
| 21 | Arabinose,d | C(C(C(C(C=O)O)O)O)O | 150.15 |
| 22 | DL-Glucuronic acid | C(=O)C(C(C(C(C(=O)O)O)O)O)O | 194.16 |
| 23 | isoferulic acid | COC1=C(C=C(C=C1)C=CC(=O)O)O | 194.2 |
| 24 | Fucopyranose, L- | CC(C(C(C(C=O)O)O)O)O | 164.18 |
| 25 | Bifendate | COC1=C2C(=C(C(=C1)C(=O)OC)C3=C4C(=C(C=C3C(=O)OC)OC)OCO4)OCO2 | 418.38 |
| 26 | gamma-aminobutyric acid | C(CC(=O)O)CN | 103.14 |
| 27 | FERULIC ACID (CIS) | COC1=C(C=CC(=C1)C=CC(=O)O)O | 194.2 |
| 28 | daidzein | C1=CC(=CC=C1C2=COC3=C(C2=O)C=CC(=C3)O)O | 254.25 |
| 29 | Ononin | COC1=CC=C(C=C1)C2=COC3=C(C2=O)C=CC(=C3)OC4C(C(C(C(O4)CO)O)O)O | 430.44 |
| 30 | formononetin | COC1=CC=C(C=C1)C2=COC3=C(C2=O)C=CC(=C3)O | 268.28 |
| 31 | Soyasaponin I | CC1C(C(C(C(O1)OC2C(C(C(OC2OC3C(C(C(OC3OC4CCC5(C(C4(C)CO)CCC6(C5CC=C7C6(CCC8(C7CC(CC8O)(C)C)C)C)C)C)C(=O)O)O)O)CO)O)O)O)O)O | 943.26 |
| 32 | choline | C[N+](C)(C)CCO | 104.2 |
| 33 | GGB | C(CON=C(N)N)C(C(=O)O)N | 176.21 |
| 34 | cis-p-Coumarate | C1=CC(=CC=C1C=CC(=O)O)O | 164.17 |
| 35 | isoflavanone | C1C(C(=O)C2=CC=CC=C2O1)C3=CC=CC=C3 | 316.33 |
| 36 | Docosanoate | CCCCCCCCCCCCCCCCCCCCCC(=O)[O-] | 340.66 |
| 37 | Flavaxin | CC1=CC2=C(C=C1C)N(C3=NC(=O)NC(=O)C3=N2)CC(C(C(CO)O)O)O | 376.41 |
| 38 | astragalosideI | CC(=O)OC1C(COC(C1OC(=O)C)OC2CCC34CC35CCC6(C(C(CC6(C5CC(C4C2(C)C)OC7C(C(C(C(O7)CO)O)O)O)C)O)C8(CCC(O8)C(C)(C)O)C)C)O | 869.17 |
| 39 | astragalosideⅢ | CC1(C(CCC23C1C(CC4C2(C3)CCC5(C4(CC(C5C6(CCC(O6)C(C)(C)O)C)O)C)C)O)OC7C(C(C(CO7)O)O)OC8C(C(C(C(O8)CO)O)O)O)C | 785.09 |
| 40 | AstragalosideIV | CC1(C(CCC23C1C(CC4C2(C3)CCC5(C4(CC(C5C6(CCC(O6)C(C)(C)O)C)O)C)C)OC7C(C(C(C(O7)CO)O)O)O)OC8C(C(C(CO8)O)O)O)C | 785.09 |
| 41 | Astraisoflavanin | COC1=C(C(=C(C=C1)C2CC3=C(C=C(C=C3)OC4C(C(C(C(O4)CO)O)O)O)OC2)OC)O | 464.51 |
| 42 | Mucronulatol | COC1=C(C(=C(C=C1)C2CC3=C(C=C(C=C3)O)OC2)OC)O | 302.35 |
| 43 | astrachrysoside A | CC1C(C(C(C(O1)OC2C(C(COC2OC3CCC45CC46CCC7(C(C(CC7(C6CC(C5C3(C)C)O)C)O)C8(CCC(O8)C(C)(C)O)C)C)O)O)O)O)O | 769.09 |
| 44 | Caffeate | C1=CC(=C(C=C1C=CC(=O)O)O)O | 180.17 |
| 45 | rutin | CC1C(C(C(C(O1)OCC2C(C(C(C(O2)OC3=C(OC4=CC(=CC(=C4C3=O)O)O)C5=CC(=C(C=C5)O)O)O)O)O)O)O)O | 610.57 |
| 46 | Lariciresinol | COC1=C(C=CC(=C1)CC2COC(C2CO)C3=CC(=C(C=C3)O)OC)O | 360.44 |
| 47 | 3'-Hydroxy-4'-methoxyisoflavone-7-O-beta-D-glucoside | COC1=C(C=C(C=C1)C2=COC3=C(C2=O)C=CC(=C3)OC4C(C(C(C(O4)CO)O)O)O)O | 446.44 |
| 48 | astrasieversianin XV | CC1C(C(C(C(O1)OC2C(C(COC2OC3CCC45CC46CCC7(C(C(CC7(C6CC(C5C3(C)C)OC8C(C(C(CO8)O)O)O)C)O)C9(CCC(O9)C(C)(C)O)C)C)O)O)O)O)O | 901.22 |
| 49 | nicotinic acid | C1=CC(=CN=C1)C(=O)O | 123.12 |
| 50 | kaempferol | C1=CC(=CC=C1C2=C(C(=O)C3=C(C=C(C=C3O2)O)O)O)O | 286.25 |
| 51 | rhamnocitrin-3-O-glucoside | COC1=CC(=C2C(=C1)OC(=C(C2=O)OC3C(C(C(C(O3)CO)O)O)O)C4=CC=C(C=C4)O)O | 462.44 |
| 52 | alpha-L-Rhamnose | CC1C(C(C(C(O1)O)O)O)O | 164.18 |
| 53 | Crystal VI | C(C(C(=O)O)N)C(=O)N | 132.14 |
| 54 | betaine | C[N+](C)(C)CC(=O)[O-] | 117.17 |
| 55 | coumarin | C1=CC=C2C(=C1)C=CC(=O)O2 | 146.15 |
| 56 | linolenic acid | CCC=CCC=CCC=CCCCCCCCC(=O)O | 278.48 |
| 57 | acetylastragaloside I | CC(=O)OC1COC(C(C1OC(=O)C)OC(=O)C)OC2CCC34CC35CCC6(C(C(CC6(C5CC(C4C2(C)C)OC7C(C(C(C(O7)CO)O)O)O)C)O)C8(CCC(O8)C(C)(C)O)C)C | 911.21 |
| 58 | (Z)-1-(2,4-dihydroxyphenyl)-3-(4-hydroxyphenyl)prop-2-en-1-one | C1=CC(=CC=C1C=CC(=O)C2=C(C=C(C=C2)O)O)O | 256.27 |
| 59 | Hirsutrin | C1=CC(=C(C=C1C2=C(C(=O)C3=C(C=C(C=C3O2)O)O)OC4C(C(C(C(O4)CO)O)O)O)O)O | 464.41 |
| 60 | (3R)-3-(2-hydroxy-3,4-dimethoxyphenyl)chroman-7-ol | COC1=C(C(=C(C=C1)C2CC3=C(C=C(C=C3)O)OC2)O)OC | 302.35 |
| 61 | LUPENONE | CC(=C)C1CCC2(C1C3CCC4C5(CCC(=O)C(C5CCC4(C3(CC2)C)C)(C)C)C)C | 424.78 |
| 62 | 3,9-dimethoxy-6H-[1]benzofuro[3,2-c]chromene-1,7-diol | COC1=CC(=C2C(=C1)OCC3=C2OC4=CC(=CC(=C34)O)OC)O | 314.31 |
| 63 | DL-Arginine | C(CC(C(=O)O)N)CN=C(N)N | 174.24 |
| 64 | Prolinum | C1CC(NC1)C(=O)O | 115.15 |
| 65 | palmitic acid | CCCCCCCCCCCCCCCC(=O)O | 256.48 |
| 66 | quercetin | C1=CC(=C(C=C1C2=C(C(=O)C3=C(C=C(C=C3O2)O)O)O)O)O | 302.25 |

**Table S4** Chemical properties of potential bioactive ingredients

| **compouds** | **counts** | **mw** | **AlogP** | **Hdon** | **Hacc** | **OB%** | **Caco-2** | **BBB** | **DL** | **FASA-** | **TPSA** | **RBN** | **HL** | **Druggability**  **(Lipinski’s rule)** |
| --- | --- | --- | --- | --- | --- | --- | --- | --- | --- | --- | --- | --- | --- | --- |
| **daidzein** | **10** | **254.25** | **2.33** | **2** | **4** | **19.44** | **0.58** | **-0.22** | **0.18** | **70.66** | **0** | **1** | **0** | **no** |
| **7-O-methylisomucronulatol** | **8** | **316.38** | **3.37** | **1** | **5** | **74.68** | **1.08** | **0.84** | **0.29** | **57.15** | **0** | **4** | **2.98** | **no** |
| **9,10-dimethoxypterocarpan-3-O-beta-D-glucoside** | **8** | **462.49** | **0.73** | **4** | **10** | **36.73** | **-0.62** | **-1.49** | **0.92** | **136.3** | **0** | **5** | **13.06** | **no** |
| **Hirsutrin** | **8** | **464.41** | **-0.58** | **8** | **12** | **1.85** | **-1.65** | **-2.3** | **0.76** | **210.5** | **0** | **4** | **0** | **no** |
| **Linoleic Acid** | **8** | **280.5** | **6.39** | **1** | **2** | **41.9** | **1.16** | **0.9** | **0.14** | **37.29** | **0.25** | **14** | **7.49** | **no** |
| **linolenic acid** | **8** | **278.48** | **5.94** | **1** | **2** | **45** | **1.21** | **0.95** | **0.14** | **37.29** | **0.26** | **13** | **6.06** | **no** |
| **(2S)-4-methoxy-7-methyl-2-[2-[(2S,3R,4S,5S,6R)-3,4,5-trihydroxy-6-(hydroxymethyl)oxan-2-yl]oxypropan-2-yl]-2,3-dihydrofuro[3,2-g]chromen-5-one** | **7** | **452.5** | **0.26** | **4** | **10** | **5.37** | **-0.79** | **-1.13** | **0.8** | **148.05** | **0** | **5** | **0** | **no** |
| **Bifendate** | **7** | **418.38** | **2.56** | **0** | **10** | **31.09** | **0.15** | **-0.06** | **0.66** | **107.98** | **0** | **7** | **17.96** | **no** |
| **formononetin** | **7** | **268.28** | **2.58** | **1** | **4** | **69.67** | **0.78** | **0.02** | **0.21** | **59.66** | **0** | **2** | **17.03** | **yes** |
| **isoferulic acid** | **7** | **194.2** | **1.61** | **2** | **4** | **50.82** | **0.49** | **0** | **0.05** | **66.76** | **0** | **3** | **2.44** | **no** |
| **isorhamnetin** | **7** | **316.28** | **1.75** | **4** | **7** | **49.6** | **0.3** | **-0.54** | **0.3** | **120.36** | **0.32** | **2** | **14.33** | **yes** |
| **Lariciresinol** | **7** | **360.44** | **2.46** | **3** | **6** | **5.52** | **0.27** | **-0.49** | **0.37** | **88.37** | **0** | **6** | **0** | **no** |
| **Ononin** | **7** | **430.44** | **0.67** | **4** | **9** | **11.52** | **-0.74** | **-1.66** | **0.77** | **138.82** | **0** | **5** | **0** | **no** |
| **quercetin** | **7** | **302.25** | **1.5** | **5** | **7** | **46.43** | **0.04** | **-0.76** | **0.27** | **131.36** | **0.38** | **1** | **14.4** | **no** |
| **rutin** | **7** | **610.57** | **-1.44** | **10** | **16** | **3.2** | **-1.92** | **-2.75** | **0.68** | **269.42** | **0** | **6** | **0** | **no** |
| **vanillic acid** | **7** | **168.16** | **1.15** | **2** | **4** | **35.47** | **0.43** | **0.09** | **0.04** | **66.76** | **0.34** | **2** | **11.62** | **no** |
| **3,9-di-O-methylnissolin** | **6** | **314.36** | **2.89** | **0** | **5** | **53.74** | **1.17** | **0.63** | **0.47** | **46.15** | **0** | **3** | **8.99** | **no** |
| **astrachrysoside A** | **6** | **314.36** | **2.89** | **0** | **5** | **53.74** | **1.17** | **0.63** | **0.47** | **46.15** | **0** | **3** | **8.99** | **no** |
| **Caffeate** | **6** | **180.17** | **1.36** | **3** | **4** | **54.97** | **0.26** | **0.1** | **0.05** | **77.76** | **0** | **2** | **1.63** | **no** |
| **choline** | **6** | **104.2** | **-1.57** | **1** | **1** | **0.47** | **0.86** | **0.64** | **0** | **20.22** | **0** | **2** | **0** | **no** |
| **coumarin** | **6** | **146.15** | **1.9** | **0** | **2** | **29.16** | **1.2** | **1.3** | **0.04** | **30.2** | **0** | **0** | **0** | **no** |
| **Jaranol** | **6** | **314.31** | **2.08** | **2** | **6** | **50.82** | **0.6** | **-0.21** | **0.29** | **89.12** | **0.28** | **3** | **15.5** | **yes** |
| **kaempferol** | **6** | **286.25** | **1.77** | **4** | **6** | **41.88** | **0.26** | **-0.55** | **0.24** | **111.12** | **0** | **1** | **14.74** | **yes** |

**Table S5** Targets of jaranol predicted using the SwissTargetPrediction Database

| **Target** | **Common name** | **Uniprot ID** | **Target Class** | **Probability*** |
| --- | --- | --- | --- | --- |
| Aldose reductase (by homology) | AKR1B1 | P15121 | Enzyme | 0.443225384 |
| Adenosine A1 receptor (by homology) | ADORA1 | P30542 | Family A G protein-coupled receptor | 0.418208027 |
| Adenosine A2a receptor (by homology) | ADORA2A | P29274 | Family A G protein-coupled receptor | 0.318252677 |
| NADPH oxidase 4 | NOX4 | Q9NPH5 | Enzyme | 0.201601849 |
| P-glycoprotein 1 | ABCB1 | P08183 | Primary active transporter | 0.184930052 |
| Induced myeloid leukemia cell differentiation protein Mcl-1 | MCL1 | Q07820 | Other cytosolic protein | 0.184930052 |
| ATP-binding cassette sub-family G member 2 | ABCG2 | Q9UNQ0 | Primary active transporter | 0.143269257 |
| Estrogen receptor beta | ESR2 | Q92731 | Nuclear receptor | 0.134939009 |
| Solute carrier family 22 member 12 | SLC22A12 | Q96S37 | Electrochemical transporter | 0.126610169 |
| Delta opioid receptor | OPRD1 | P41143 | Family A G protein-coupled receptor | 0.126610169 |
| Estradiol 17-beta-dehydrogenase 2 | HSD17B2 | P37059 | Enzyme | 0.118277085 |
| Estradiol 17-beta-dehydrogenase 1 | HSD17B1 | P14061 | Enzyme | 0.118277085 |
| Tyrosine-protein kinase receptor FLT3 | FLT3 | P36888 | Kinase | 0.118277085 |
| Beta-secretase 1 | BACE1 | P56817 | Protease | 0.118277085 |
| Cytochrome P450 1B1 | CYP1B1 | Q16678 | Cytochrome P450 | 0.118277085 |
| Adenosine A3 receptor | ADORA3 | P0DMS8 | Family A G protein-coupled receptor | 0.118277085 |
| Beta amyloid A4 protein | APP | P05067 | Membrane receptor | 0.118277085 |
| Serine/threonine-protein kinase PIM1 | PIM1 | P11309 | Kinase | 0.10994577 |
| Plasminogen | PLG | P00747 | Protease | 0.10994577 |
| Epidermal growth factor receptor erbB1 | EGFR | P00533 | Kinase | 0.10994577 |
| Stem cell growth factor receptor | KIT | P10721 | Kinase | 0.10994577 |
| PI3-kinase p110-gamma subunit | PIK3CG | P48736 | Enzyme | 0.10994577 |
| ALK tyrosine kinase receptor | ALK | Q9UM73 | Kinase | 0.10994577 |
| Xanthine dehydrogenase | XDH | P47989 | Oxidoreductase | 0.101613855 |
| Nitric oxide synthase, inducible (by homology) | NOS2 | P35228 | Enzyme | 0.101613855 |
| Cyclooxygenase-2 (by homology) | PTGS2 | P35354 | Oxidoreductase | 0.101613855 |
| Mu opioid receptor | OPRM1 | P35372 | Family A G protein-coupled receptor | 0.101613855 |
| Carbonic anhydrase II | CA2 | P00918 | Lyase | 0.101613855 |
| Carbonic anhydrase VII | CA7 | P43166 | Lyase | 0.101613855 |
| Carbonic anhydrase XII | CA12 | O43570 | Lyase | 0.101613855 |
| Microtubule-associated protein tau | MAPT | P10636 | Unclassified protein | 0.101613855 |
| DNA topoisomerase II alpha | TOP2A | P11388 | Isomerase | 0.101613855 |
| Insulin receptor | INSR | P06213 | Kinase | 0.101613855 |
| Myosin light chain kinase, smooth muscle | MYLK | Q15746 | Kinase | 0.101613855 |
| DNA-(apurinic or apyrimidinic site) lyase | APEX1 | P27695 | Enzyme | 0.101613855 |
| Estrogen receptor alpha | ESR1 | P03372 | Nuclear receptor | 0.101613855 |
| Serine/threonine-protein kinase AKT | AKT1 | P31749 | Kinase | 0.101613855 |
| Arachidonate 5-lipoxygenase | ALOX5 | P09917 | Oxidoreductase | 0.101613855 |
| Ornithine decarboxylase | ODC1 | P11926 | Lyase | 0.101613855 |
| Matrix metalloproteinase 9 | MMP9 | P14780 | Protease | 0.101613855 |
| Matrix metalloproteinase 2 | MMP2 | P08253 | Protease | 0.101613855 |
| Carbonic anhydrase IV | CA4 | P22748 | Lyase | 0.101613855 |
| Hepatocyte growth factor receptor | MET | P08581 | Kinase | 0.101613855 |
| Multidrug resistance-associated protein 1 | ABCC1 | P33527 | Primary active transporter | 0.101613855 |
| Insulin-like growth factor I receptor | IGF1R | P08069 | Kinase | 0.101613855 |
| Vasopressin V2 receptor | AVPR2 | P30518 | Family A G protein-coupled receptor | 0.101613855 |
| Interleukin-8 receptor A | CXCR1 | P25024 | Family A G protein-coupled receptor | 0.101613855 |
| Arachidonate 15-lipoxygenase | ALOX15 | P16050 | Enzyme | 0.101613855 |
| Arachidonate 12-lipoxygenase | ALOX12 | P18054 | Enzyme | 0.101613855 |
| Tyrosinase | TYR | P14679 | Oxidoreductase | 0.101613855 |
| Aryl hydrocarbon receptor | AHR | P35869 | Transcription factor | 0.101613855 |
| Estrogen-related receptor alpha | ESRRA | P11474 | Nuclear receptor | 0.101613855 |
| Cytochrome P450 19A1 | CYP19A1 | P11511 | Cytochrome P450 | 0.101613855 |
| Carbonic anhydrase I | CA1 | P00915 | Lyase | 0.101613855 |
| Carbonic anhydrase VI | CA6 | P23280 | Lyase | 0.101613855 |
| Carbonic anhydrase IX | CA9 | Q16790 | Lyase | 0.101613855 |
| NEDD8-activating enzyme E1 regulatory subunit | NAE1 | Q13564 | Unclassified protein | 0.101613855 |
| Thrombin | F2 | P00734 | Protease | 0.101613855 |
| Cyclin-dependent kinase 1 | CDK1 | P06493 | Kinase | 0.101613855 |
| G-protein coupled receptor 35 | GPR35 | Q9HC97 | Family A G protein-coupled receptor | 0.101613855 |
| Glycogen synthase kinase-3 beta | GSK3B | P49841 | Kinase | 0.101613855 |
| Receptor-type tyrosine-protein phosphatase S | PTPRS | Q13332 | Phosphatase | 0.101613855 |
| Lysine-specific demethylase 4D-like | KDM4E | B2RXH2 | Eraser | 0.101613855 |
| Monoamine oxidase A | MAOA | P21397 | Oxidoreductase | 0.101613855 |
| Acetylcholinesterase | ACHE | P22303 | Hydrolase | 0.101613855 |
| Telomerase reverse transcriptase | TERT | O14746 | Enzyme | 0.101613855 |
| Serine/threonine-protein kinase Aurora-B | AURKB | Q96GD4 | Kinase | 0.101613855 |
| Tyrosine-protein kinase SRC | SRC | P12931 | Kinase | 0.101613855 |
| Focal adhesion kinase 1 | PTK2 | Q05397 | Kinase | 0.101613855 |
| Vascular endothelial growth factor receptor 2 | KDR | P35968 | Kinase | 0.101613855 |
| Serine/threonine-protein kinase PLK1 | PLK1 | P53350 | Kinase | 0.101613855 |
| Protein kinase N1 | PKN1 | Q16512 | Kinase | 0.101613855 |
| Serine/threonine-protein kinase NEK2 | NEK2 | P51955 | Kinase | 0.101613855 |
| Serine/threonine-protein kinase NEK6 | NEK6 | Q9HC98 | Kinase | 0.101613855 |
| Tyrosine-protein kinase receptor UFO | AXL | P30530 | Kinase | 0.101613855 |
| NUAK family SNF1-like kinase 1 | NUAK1 | O60285 | Kinase | 0.101613855 |
| Cyclin-dependent kinase 5/CDK5 activator 1 | CDK5R1 CDK5 | Q15078 Q00535 | Kinase | 0.101613855 |
| Cyclin-dependent kinase 1/cyclin B | CCNB3 CDK1 CCNB1 CCNB2 | Q8WWL7 P06493 P14635 O95067 | Other cytosolic protein | 0.101613855 |
| Cyclin-dependent kinase 6 | CDK6 | Q00534 | Kinase | 0.101613855 |
| Beta-galactoside alpha-2,6-sialyltransferase 1 | ST6GAL1 | P15907 | Transferase | 0.101613855 |
| Voltage-gated potassium channel subunit Kv1.3 | KCNA3 | P22001 | Voltage-gated ion channel | 0.101613855 |
| Phosphodiesterase 5A | PDE5A | O76074 | Phosphodiesterase | 0.101613855 |
| Tyrosine-protein kinase LCK | LCK | P06239 | Kinase | 0.101613855 |
| Tyrosine-protein kinase SYK | SYK | P43405 | Kinase | 0.101613855 |
| Casein kinase II alpha | CSNK2A1 | P68400 | Kinase | 0.101613855 |
| Endoplasmin | HSP90B1 | P14625 | Other membrane protein | 0.101613855 |
| Butyrylcholinesterase | BCHE | P06276 | Hydrolase | 0.101613855 |
| Phospholipase A2 group IIA | PLA2G2A | P14555 | Enzyme | 0.101613855 |
| Death-associated protein kinase 1 | DAPK1 | P53355 | Kinase | 0.101613855 |
| DNA-3-methyladenine glycosylase | MPG | P29372 | Enzyme | 0.101613855 |
| Cytochrome P450 1A1 | CYP1A1 | P04798 | Cytochrome P450 | 0.101613855 |
| Cytochrome P450 1A2 | CYP1A2 | P05177 | Cytochrome P450 | 0.101613855 |
| CaM kinase II beta | CAMK2B | Q13554 | Kinase | 0.101613855 |
| G protein-coupled receptor kinase 6 | GRK6 | P43250 | Kinase | 0.101613855 |
| Cyclin-dependent kinase 2 | CDK2 | P24941 | Kinase | 0.101613855 |
| 6-phosphofructo-2-kinase/fructose-2,6-bisphosphatase 3 | PFKFB3 | Q16875 | Enzyme | 0.101613855 |
| Glucagon receptor | GCGR | P47871 | Family B G protein-coupled receptor | 0.101613855 |
| Dopamine D4 receptor | DRD4 | P21917 | Family A G protein-coupled receptor | 0.101613855 |
| Glyoxalase I | GLO1 | Q04760 | Enzyme | 0.101613855 |
| AMY1C | AMY1A | P04745 | Enzyme | 0.101613855 |

**Table S6** Content of jaranol in different batches of *Astragalus membranaceus*

| **Batches** | **Contents (ug/g）** | **Batches** | **Contents (ug/g）** |
| --- | --- | --- | --- |
| 1 | 0.0255 | 9 | 0.0731 |
| 2 | 0.0096 | 10 | 0.0433 |
| 3 | 0.1262 | 11 | 0.0356 |
| 4 | 0.0163 | 12 | 0.0522 |
| 5 | 0.0168 | 13 | 0.0416 |
| 6 | 0.0444 | 14 | 0.0469 |
| 7 | 0.0594 | 15 | 0.0469 |
| 8 | 0.0337 | 16 | 0.0408 |

**Table S7** Structural and functional parameters of the mouse heart

| **parameters** | **LVPW;d** | **LVID;d** | **LVEF%** | **LVFS%** | **E/A** | **E/E'** |
| --- | --- | --- | --- | --- | --- | --- |
| **Vehicle** | **0.66±0.08** | **4.13±0.18** | **66.71±6.86** | **36.03±4.21** | **1.43±0.32** | **22.86±4.62** |
| **TAC** | **0.98±0.14^***^** | **4.31±0.33** | **49.59±9.11^***^** | **25.21±5.14^***^** | **1.02±0.27^**^** | **42.47±11.04^***^** |
| **TAC+Jaranol** | **0.81±0.16^#^** | **4.2±0.42** | **59.52±8.56^#^** | **31.23±5.5^#^** | **1.3±0.17^#^** | **28.72±7.57^##^** |

Data are presented as the mean±SD, 1-way ANOVA with Tukey post *hoc* test, compared to Vehicle, ^**^*P*<0.01, ^***^*P*<0.001, compared to TAC, ^#^*P*<0.05, ^##^*P*<0.01.

**Table S8** The promoter sequence of the human TLR2 gene

| Upstream by 2000 bases of coding sequence | aaactatgaatatatacaatgaaagttaccctttccccaaatattagtac  atttcatatgccgtgttgattttctaactttaatcaacatttttcttttt  tttggtagagagttataggaggtcttatatctagatacagaaggagactt  ggagtctaacctgggatttgccacatgttaactgtgagccatgcgactct  gggcaagttccctaatcactttaaccaaaggaatagtaatatcagaatta  tttattgcatgtgttattgtaagattcagatgaaattctagatgtgaagg  tgcttcaaaaatgtcaagaactaggccgggtgctgtggctcacacctgta  atcccagcactttgggaggcctaggctggtggatcacttggtgccaggag  ttcaagaccagcctggtcaacatagcaaaaccctgtctctaccaaaaata  caaaaattagccaggcgtggtggcacgcacctgtaatcccacctacttgg  gaggctgaggcagagaattgcttgaatctgggaggtggagactgcagtga  gatgagattgcaccactgcactccagcctgggtgacaaaacgagactcca  tctcagaaaaaaaaaaaaaaaaaaagaaaatgtcaagaactgaacaagta  taaggactgtgtctgaattggacgtgctaatagctttgtggacagtgtac  aactaccttcaatatatgcagaggacactgatttgtggctaaataaatga  tttctatctgactttgccagacaattctaattgactttgggattatagag  caatagcatcaaaatccaccttaggcagttcaactagggaagagaaatag  agaaaaaacaagtttttagtttcaggagaaataatttgaaggaagttttc  ttttgtcagaaatcacaaggtagagagctggagatttggaattaataaga  aagcattttcagccagctggacaacctgatggtcaggataaagaaaaata  gtaagatagtgacctgtatacaaaaacctctcaatttcatatttaggtct  attgattaagtttccctgatgttccaaccatcagagaggattagggatgt  ggaacagggtaggagaaacagccctggatttaaggtaaaattctggtttc  aaatctcagttctgtcatatgttatacaattcacatgacattttcttgtg  taagggaggaacaaggatgcttgctctgcttcatatctcactttgtgaaa  atcaaatgtaattatgaacataaaaggtagattattaacattaaggcagt  tgaattttaaaagcttgttttcttcccttatacatattcctaaccgtgga  gacagctaggttctgtggcaatgaagaaaattgatcaatgactgtttttg  gtattactttgggaagtagggatggaataggggcaagggaagggcttagc  ttcaaactgtagacagaagtgggaagcccaaatctttgcatggtgagaag  tgggatggaatgtaggtgctagagatggtgcaccagtagggactgtcact  ggattggggaaaaaaatccggggctgtccttatctgctcccttaggcaaa  atcgttctgtgttagtctggagggtgaacctattttaatgcaggagggca  tagacttttgggtggaagcggaccctggagatgtactggcaggtgaatag  agtgtggaaaagtggggaaaggagaagcacgattcatgagcctgagttcg  ggcttccatggatggaaaagcatcaggagtggaatttaaagacggtgtgt  ttcaggtgatgtgaggtcctggatgcccgaagcttcaagaaaatactggt  tgggcacttagctttccctgtggttgccaatcccacgcaggccgcctcta  gcgtctcgattcgcttttctctgacctggaacctccgccaagcccccagc  tctcttcttcgacccagccttgcacggggcagctgtcggggcaggacccg |
| --- | --- |

**Table S9** Total 19 putative site(s) were predicted with relative profile score threshold 80% in JASPAR CORE database

| **Name** | **Score** | **Relative Score** | **Start** | **End** | **Strand** | **Predicted Sequence** |
| --- | --- | --- | --- | --- | --- | --- |
| MA1559.2.SNAI3 | 10.968436 | 0.9274057 | 1689 | 1697 | + | gcaggtgaa |
| MA1559.2.SNAI3 | 4.918226 | 0.83054096 | 1512 | 1520 | + | gtaggtgct |
| MA1559.2.SNAI3 | 4.865062 | 0.82968986 | 375 | 383 | + | gctggtgga |
| MA1559.2.SNAI3 | 4.672295 | 0.8266036 | 531 | 539 | + | ggaggtgga |
| MA1559.2.SNAI3 | 4.5702024 | 0.82496905 | 296 | 304 | + | gaaggtgct |
| MA1559.2.SNAI3 | 4.4072204 | 0.8223597 | 1802 | 1810 | + | tcaggtgat |
| MA1559.2.SNAI3 | 4.132612 | 0.81796324 | 257 | 265 | + | gcatgtgtt |
| MA1559.2.SNAI3 | 3.7225504 | 0.811398 | 1979 | 1987 | + | gcagctgtc |
| MA1559.2.SNAI3 | 3.2604764 | 0.80400014 | 644 | 652 | + | acaagtata |
| MA1559.2.SNAI3 | 3.1083455 | 0.8015645 | 1640 | 1648 | + | gcaggaggg |
| MA1559.1.SNAI3 | 10.934558 | 0.9152495 | 1688 | 1697 | + | ggcaggtgaa |
| MA1559.1.SNAI3 | 4.8243017 | 0.82071894 | 374 | 383 | + | ggctggtgga |
| MA1559.1.SNAI3 | 4.6305037 | 0.8177208 | 530 | 539 | + | gggaggtgga |
| MA1559.1.SNAI3 | 4.4304013 | 0.81462497 | 1511 | 1520 | + | tgtaggtgct |
| MA1559.1.SNAI3 | 4.167712 | 0.810561 | 643 | 652 | + | aacaagtata |
| MA1559.1.SNAI3 | 4.0815377 | 0.8092278 | 295 | 304 | + | tgaaggtgct |
| MA1559.1.SNAI3 | 3.9213216 | 0.8067491 | 1801 | 1810 | + | ttcaggtgat |
| MA1559.1.SNAI3 | 3.6844 | 0.8030837 | 1978 | 1987 | + | ggcagctgtc |
| MA1559.1.SNAI3 | 3.645146 | 0.80247647 | 256 | 265 | + | tgcatgtgtt |

**Table S10** Proteome microarray assay results

| **65 proteins** | | **34 proteins** | | **19 proteins** | |
| --- | --- | --- | --- | --- | --- |
| **Name** | **ID** | **Name** | **ID** | **Name** | **ID** |
| SPN | JHU00850.B1C2 | KJ906227 | JHU03573.B1C22 | PPP2R3B | JHU09854.B6C19 |
| FAM131C | JHU05680.B1C7 | SCAMP4 | JHU04870.B1C25 | EFHB | JHU08954.B7C8 |
| NAT9 | JHU02457.B1C8 | AK6 | JHU00087.B2C5 | ELP5 | JHU15292.B7C24 |
| DDIT4 | JHU01282.B1C27 | SHCBP1 | JHU04112.B2C16 | TMEM204 | JHU07406.B7C27 |
| GTF3C6 | JHU00785.B2C3 | UBE2D4 | JHU02391.B2C20 | RAPGEF1 | JHU07468.B7C29 |
| ZCCHC7 | JHU02874.B2C14 | ARMCX2 | JHU05383.B3C2 | CTDSP1 | JHU07422.B7C29 |
| UBE2Q2 | JHU04793.B2C22 | FAM131C | JHU00996.B3C6 | AGTR1 | JHU16058.B8C21 |
| KEAP1 | JHU02347.B2C26 | MAGEE1 | JHU06955.B5C2 | LRRC57 | JHU06870.B8C31 |
| FAM122A | JHU00995.B3C24 | AMZ2P1_frag | JHU10770.B5C3 | ZNF326 | JHU13587.B10C14 |
| TMEM263 | JHU03555.B3C32 | PPP2R3B | JHU09854.B5C4 | CASC4 | JHU13513.B10C17 |
| FAM131B | JHU05594.B4C1 | GRB2 | JHU07525.B5C6 | WNK1 | JHU13534.B10C22 |
| PYHIN1 | JHU00734.B4C10 | FOXP4 | JHU07036.B5C8 | TBL2 | JHU13573.B10C32 |
| KJ900886_frag | JHU08688.B5C4 | ALDH3B2 | JHU07397.B5C8 | TOX4 | JHU13579.B11C9 |
| ATG10 | JHU09797.B5C6 | A2ML1 | JHU10079.B5C9 | MAGEA6 | JHU13255.B11C16 |
| CLMP | JHU07014.B5C9 | IL15 | JHU10976.B5C16 | GPATCH2L | JHU13510.B11C17 |
| PPP2R3B | JHU09854.B5C16 | AGTR1 | JHU16058.B5C17 | PDE9A | JHU13549.B11C29 |
| WNT7B | JHU08733.B5C24 | PPM1M | JHU09379.B5C17 | IGIP | JHU12109.B13C24 |
| AC018359.1_frag | JHU10942.B5C29 | CDK6 | JHU07982.B5C18 | INTS4 | JHU15331.B15C28 |
| MCCC1 | JHU08487.B6C2 | QPCTL | JHU07467.B5C22 | ACACB | JHU25646.B20C15 |
| KJ904395 | JHU06911.B6C13 | ADPRHL1 | JHU10752.B5C23 |  | |
| ADAM12 | JHU07011.B6C27 | PSMD2 | JHU07165.B5C24 |  | |
| NRBP1 | JHU08980.B6C32 | ZSCAN20 | JHU09119.B5C24 |  | |
| MAGEC2 | JHU06193.B7C2 | C11orf52 | JHU07785.B5C27 |  | |
| HLCS | JHU10397.B7C2 | KJ902696 | JHU09345.B5C27 |  | |
| TPRA1 | JHU07039.B7C8 | SEC13 | JHU09387.B5C27 |  | |
| PLEKHG5 | JHU07463.B7C8 | DAB1 | JHU10095.B5C27 |  | |
| TGM1 | JHU08437.B7C10 | LCK | JHU09167.B5C31 |  | |
| CDA | JHU06835.B7C13 | GSTM4 | JHU10775.B6C2 |  | |
| TSKU | JHU07100.B7C14 | OR4D10 | JHU06392.B6C6 |  | |
| TSPAN7 | JHU07481.B7C17 | H1FX | JHU08674.B6C11 |  | |
| FXYD7 | JHU07707.B7C20 | ZNF333 | JHU08062.B6C31 |  | |
| C3orf20 | JHU06831.B7C21 | NFKB | JHU07070.B7C22 |  | |
| MAGEA9 | JHU08970.B7C22 | RIPPLY1 | JHU11968.B9C2 |  | |
| HABP2 | JHU07040.B7C29 | NACA2 | JHU12216.B9C21 |  | |
| POLR3E | JHU07465.B7C32 |  |  |  |  |
| DRC3 | JHU07726.B8C3 |  |  |  |  |
| ATP1A2 | JHU07402.B8C9 |  |  |  |  |
| LZIC | JHU10127.B8C9 |  |  |  |  |
| GALNT3 | JHU06856.B8C13 |  |  |  |  |
| CPNE5 | JHU06837.B8C18 |  |  |  |  |
| CEP41 | JHU06907.B8C21 |  |  |  |  |
| RFWD3 | JHU07082.B8C27 |  |  |  |  |
| LURAP1L | JHU06832.B8C31 |  |  |  |  |
| DDX20 | JHU06839.B8C31 |  |  |  |  |
| CBLN4 | JHU13030.B9C3 |  |  |  |  |
| DTNBP1 | JHU11635.B9C17 |  |  |  |  |
| C1orf74 | JHU13024.B9C26 |  |  |  |  |
| KCNAB1 | JHU13741.B9C28 |  |  |  |  |
| IFFO1 | JHU13149.B9C32 |  |  |  |  |
| CUL4A | JHU11631.B10C8 |  |  |  |  |
| FAM131B | JHU12373.B10C8 |  |  |  |  |
| GSTA4 | JHU13530.B10C8 |  |  |  |  |
| STAC3 | JHU13569.B10C8 |  |  |  |  |
| SUV420H1 | JHU13571.B10C9 |  |  |  |  |
| CCNA2 | JHU13705.B10C10 |  |  |  |  |
| GPR85 | JHU12386.B10C27 |  |  |  |  |
| ZNF274 | JHU13978.B10C32 |  |  |  |  |
| IGSF3 | JHU12959.B11C13 |  |  |  |  |
| ALDH9A1_frag | JHU16249.B11C27 |  |  |  |  |
| FAM131B | JHU16409.B12C12 |  |  |  |  |
| PC | JHU14030.B12C24 |  |  |  |  |
| PJA2 | JHU13367.B13C3 |  |  |  |  |
| MAGEA9 | JHU18292.B13C9 |  |  |  |  |
| PCCA | JHU03995.B14C3 |  |  |  |  |
| RNF7 | JHU02846.B16C15 |  |  |  |  |
|  |  |  |  |  |  |

*
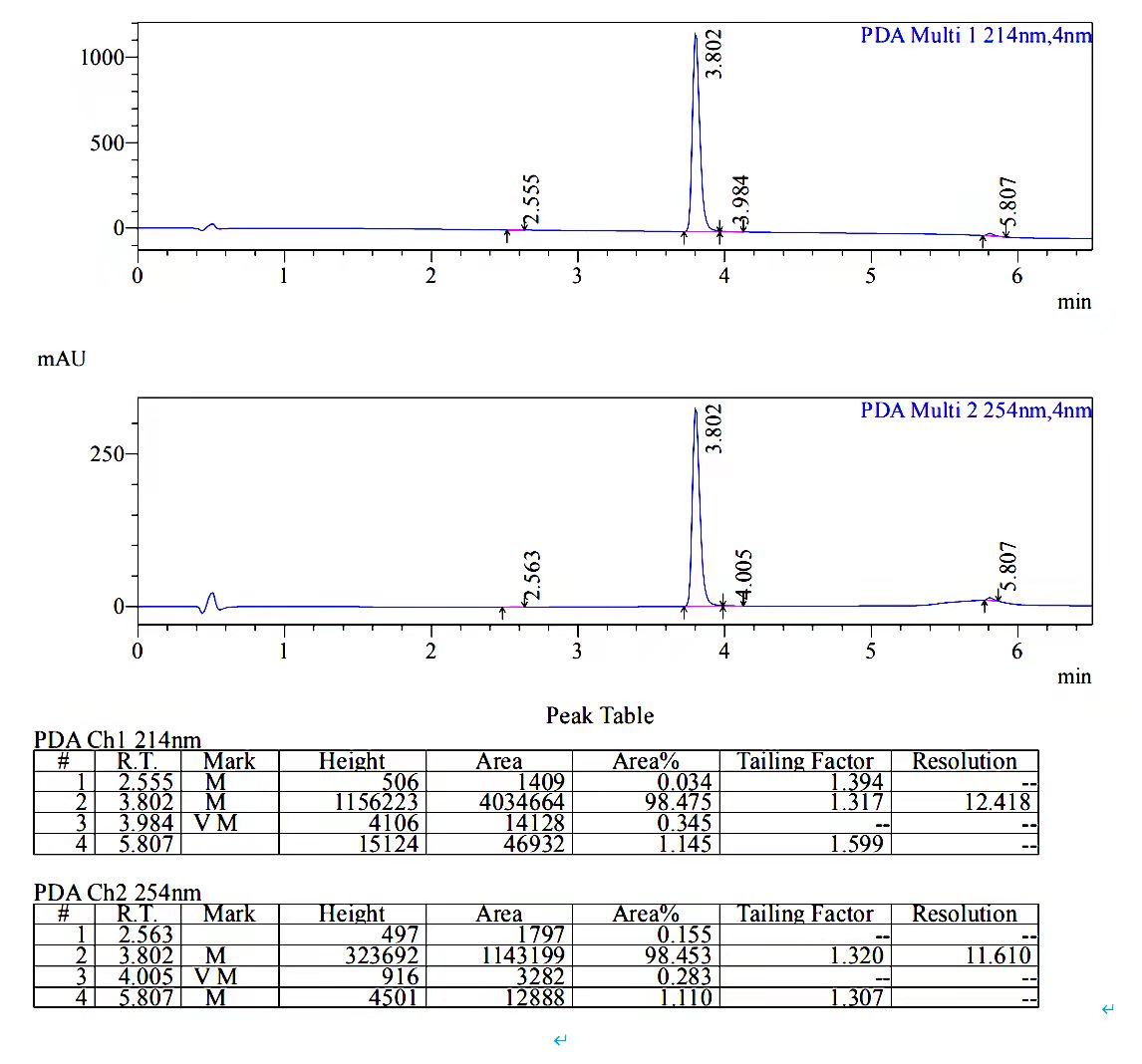
***Figure S1** Chromatograms of jaranol

*
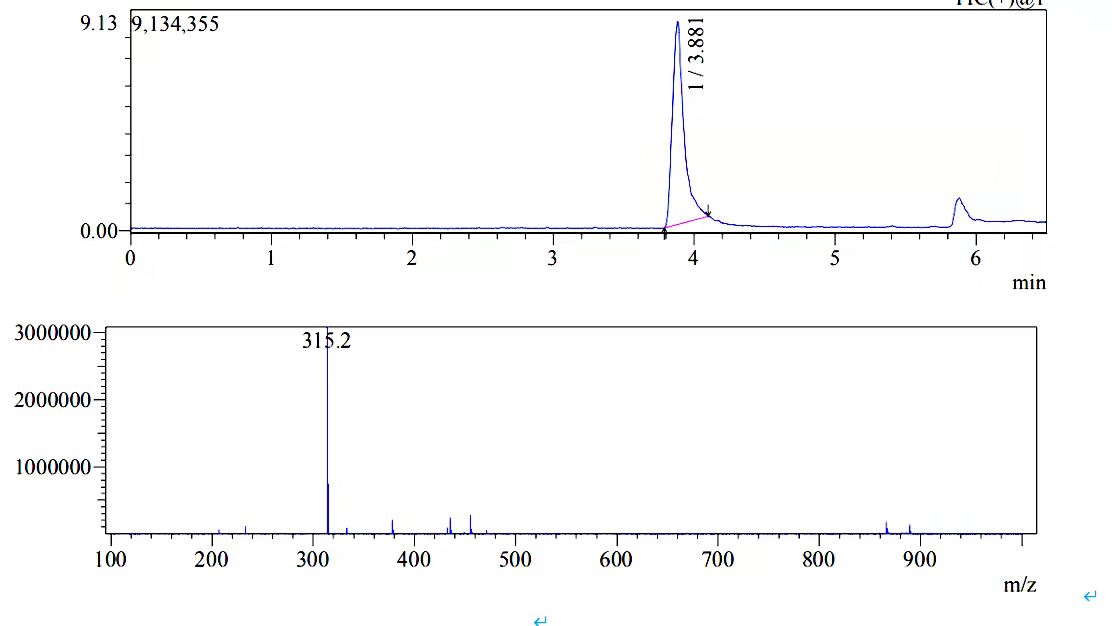
*

**Figure S2** LC/MS spectrum of jaranol


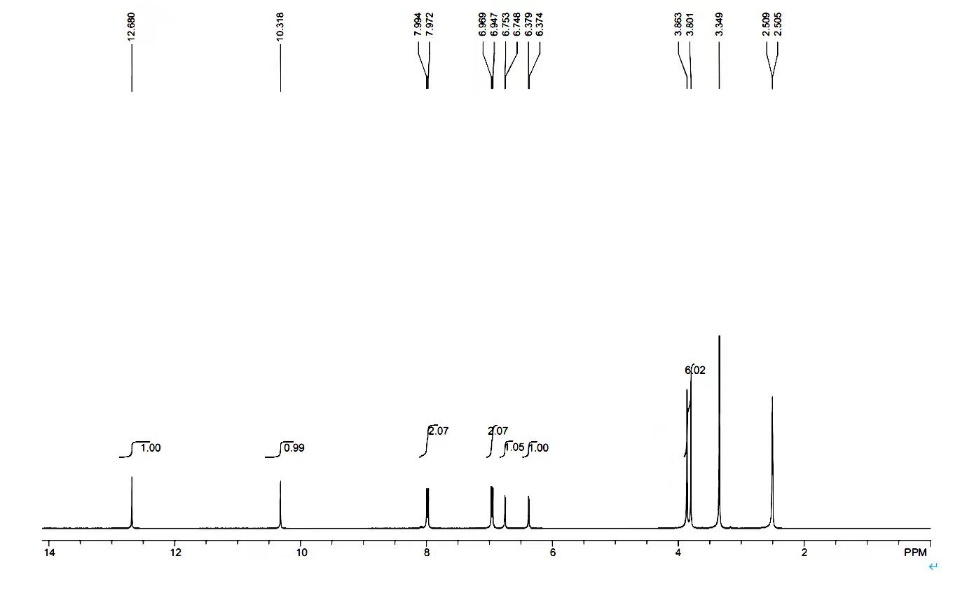


**Figure S3** Nuclear magnetic resonance (NMR) spectrum of jaranol


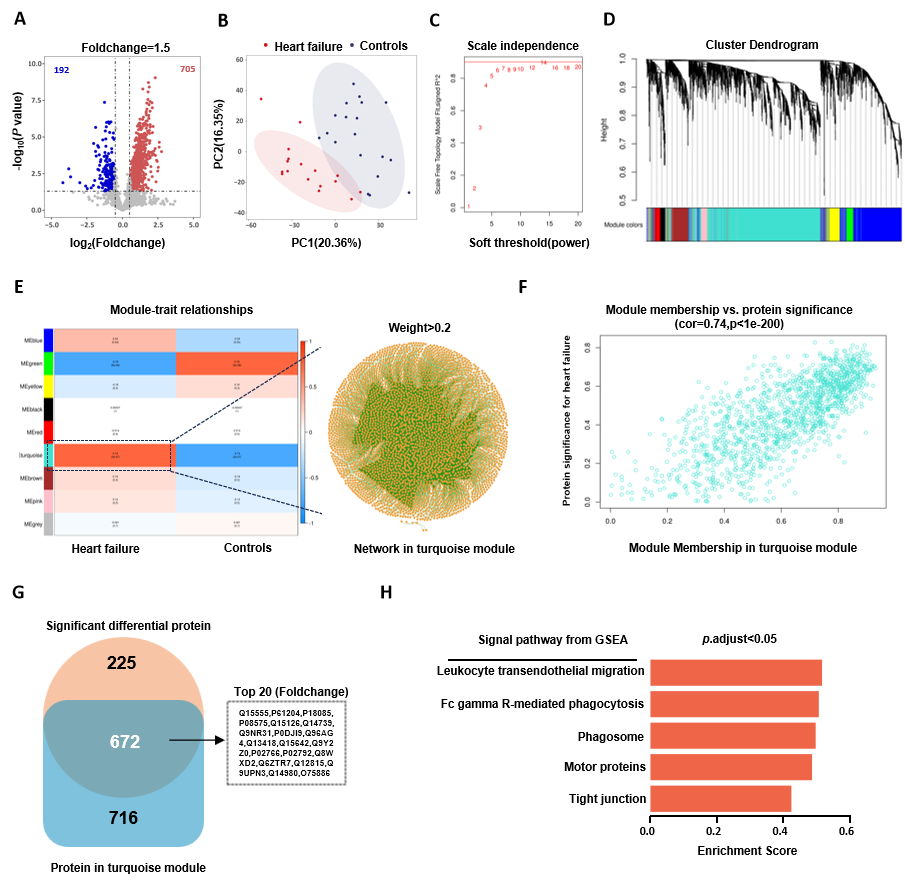


**Figure S4 Identification of crucial proteins related to cardiac remodelling through WGCNA of serum proteomic profile of patients with heart failure.** (A) Volcano plot represents the differential serum protein level between patients with heart failure and controls; (B) Unsupervised principal component analysis (PCA) plot demonstrating significant differences in proteomic profile between patients with heart failure and controls; (C) The scale-free fit index and the average connectivity of soft threshold power; (D) Identification of 9 modules presented as a clustering tree; (E) The correlation of these modules between the patients with heart failure and controls (left) and Co-expression networks corresponding to turquoise modules (right); (F) Scatter plot showed the correlation between proteins in the turquoise module and phenotype of heart failure; (G) The Venn diagram illustrated 672 proteins related to cardiac remodelling; (H) GSEA was performed on 672 proteins related to cardiac remodelling.


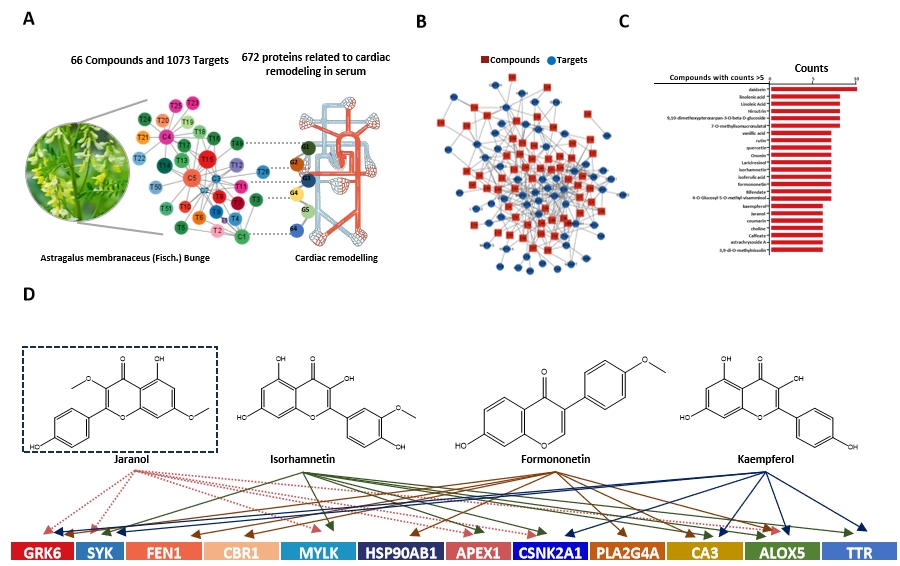


**Figure S5 Jaranol displayed the potential bioactivity on cardiac remodelling.** (A) A workflow of screening active ingredients from *Astragalus membranaceus*; (B) Network showed cardiac remodeling-related targets that regulated by ingredients from *Astragalus membranaceus*; (C) The counts of cardiac remodeling-related targets that regulated by ingredients from *Astragalus membranaceus*; (D) Jaranol displayed potential bioactivity on cardiac remodelling; (E) High-performance liquid chromatography of jaranol.


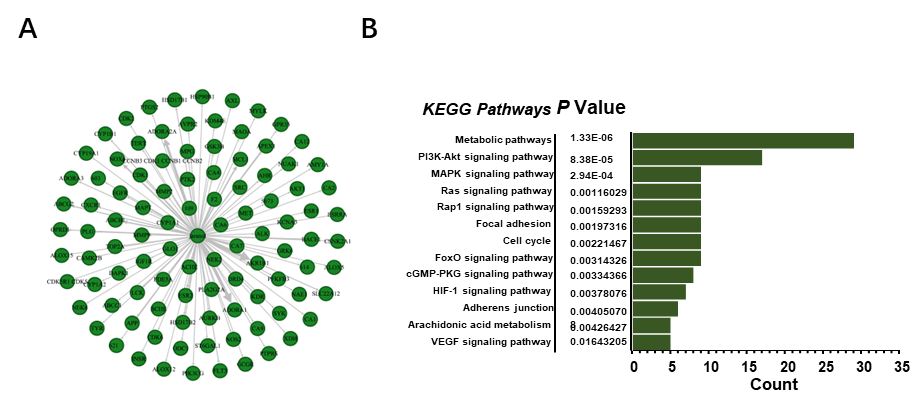


**Figure S6 The potential protective effect of jaranol against cardiac remodelling.** (A) The targets of jaranol were predicted using the SwissTargetPrediction Database. (B) Functional enrichment analysis results for the targets of jaranol.


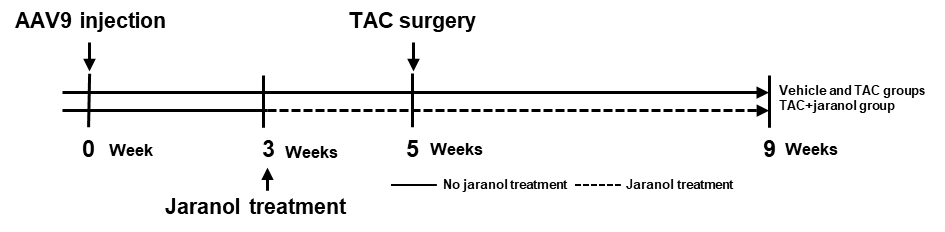


**Figure S7** Diagram of the experimental approach


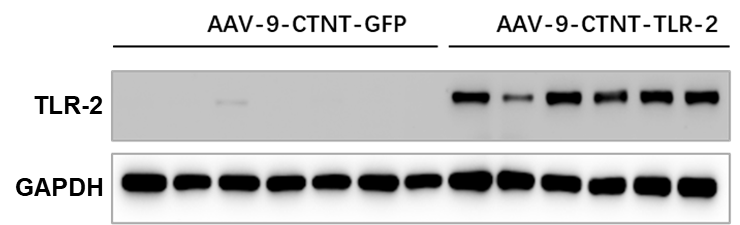


**Figure S8** TLR2 was specifically overexpressed by the AAV-9 vector in mice


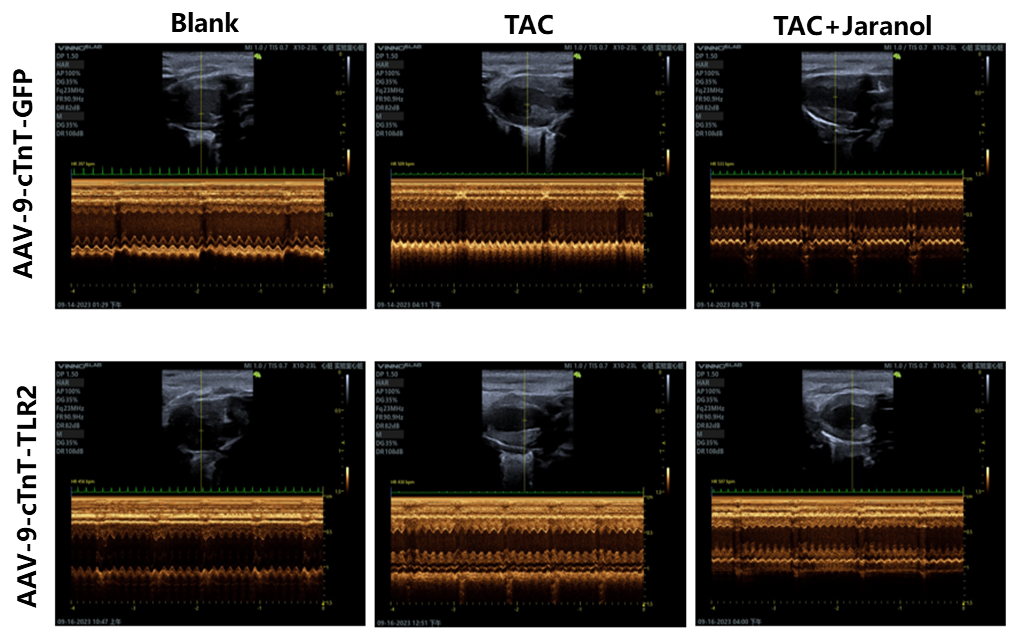


**Figure S9** Representative echocardiographic images of the mice in each experimental group.


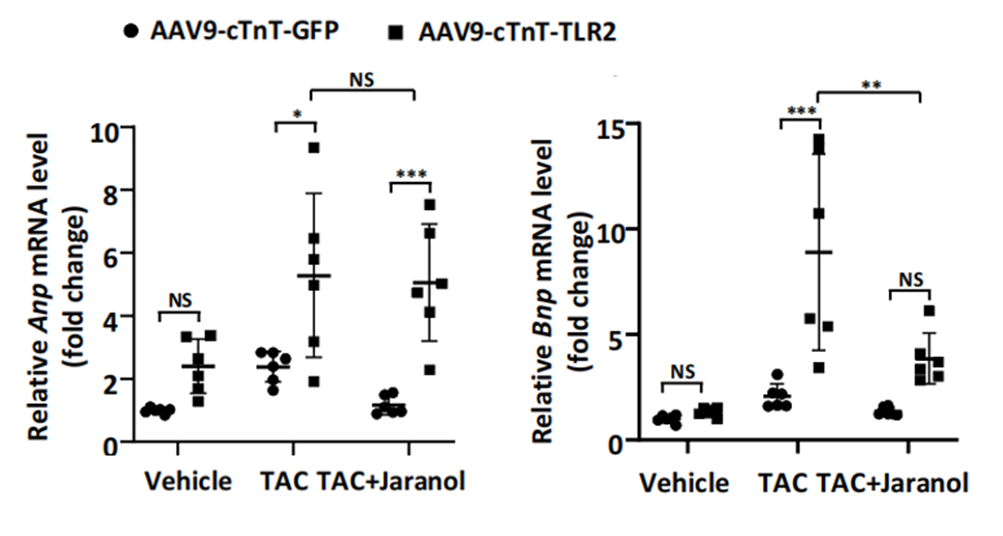


**Figure S10 mRNA levels of hypertrophy-associated genes were measured in mouse hearts.** The data are presented as means±SDs; two-way ANOVA with Tukey’s post *hoc* test; ^*^*P*<0.05, ^**^*P*<0.01, and ^***^*P*<0.001.


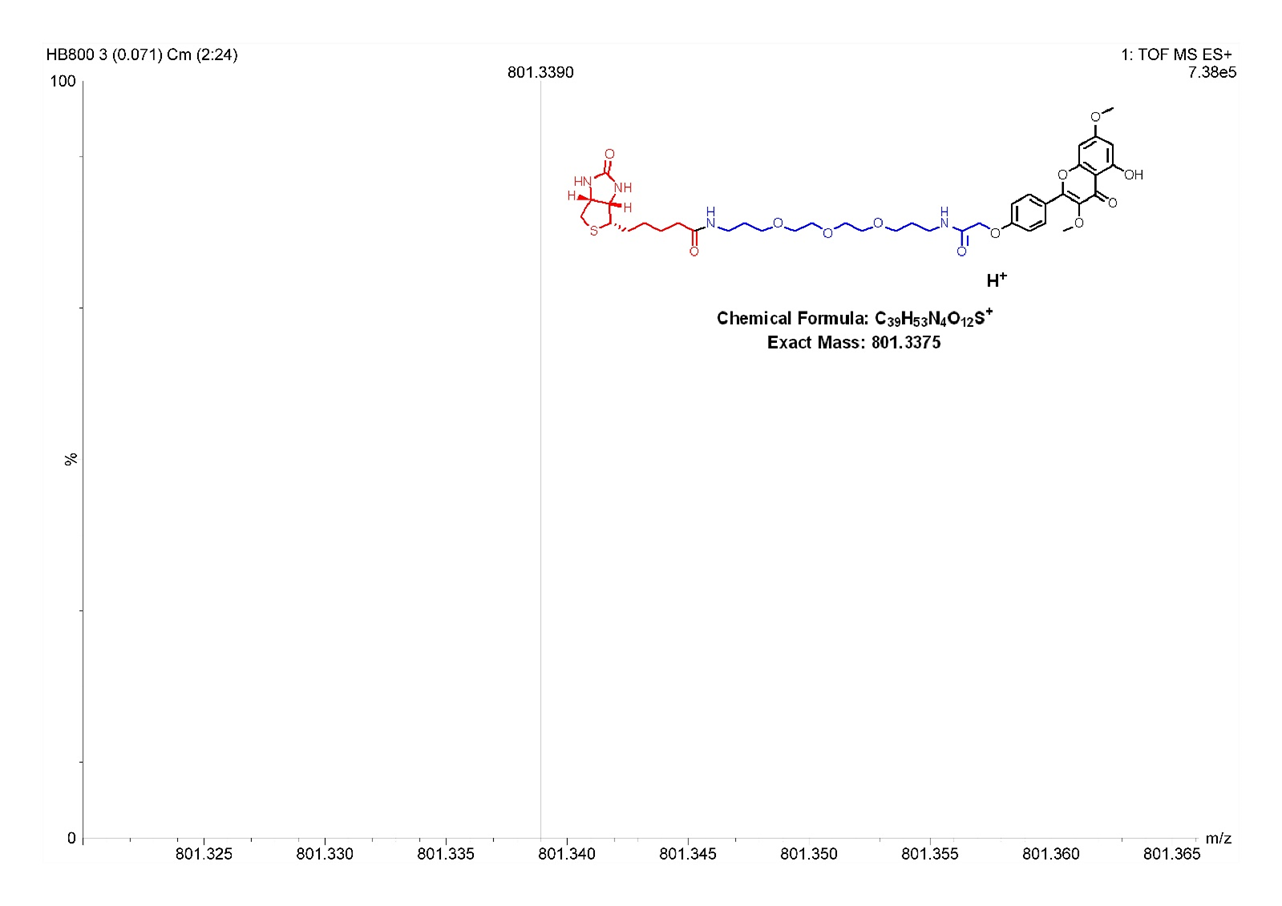


**Figure S11** Mass spectrometry of biotinylated jaranol


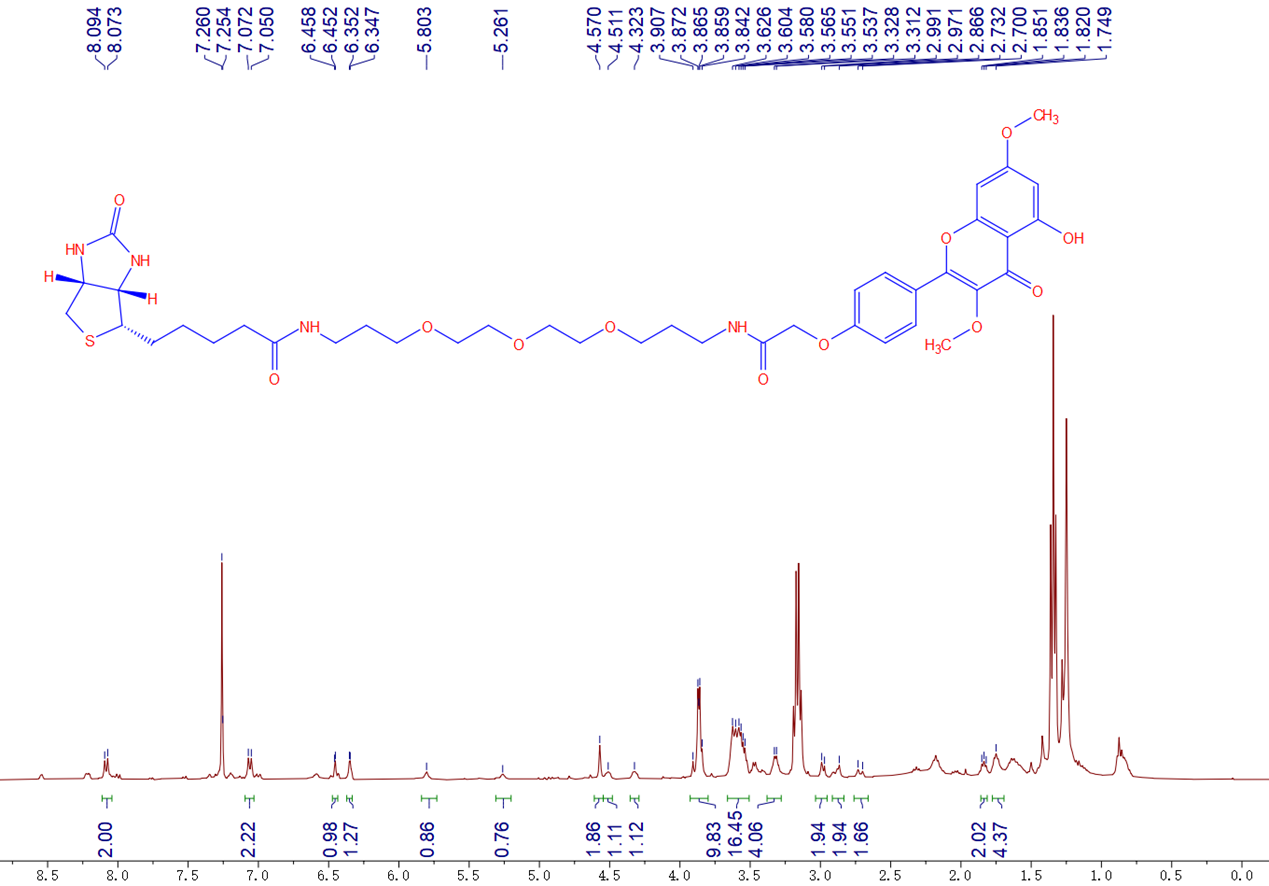


**Figure S12** Nuclear magnetic resonance (NMR) spectra of biotinylated-jaranol


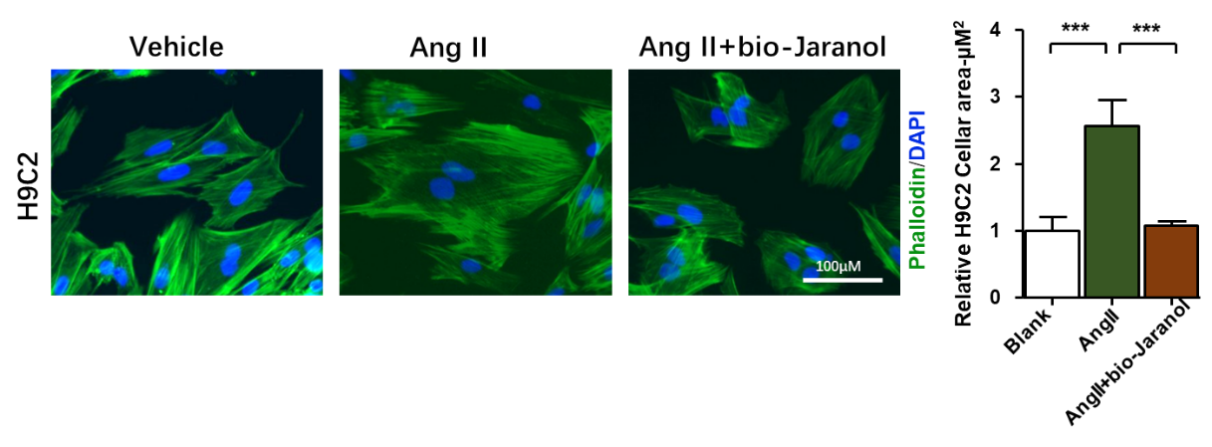


**Figure S13** The biological activity of biotinylated jaranol was evaluated in H9C2 cells. The data are presented as means±SDs; one-way ANOVA with Tukey’s post hoc test; ^***^*P*<0.001.


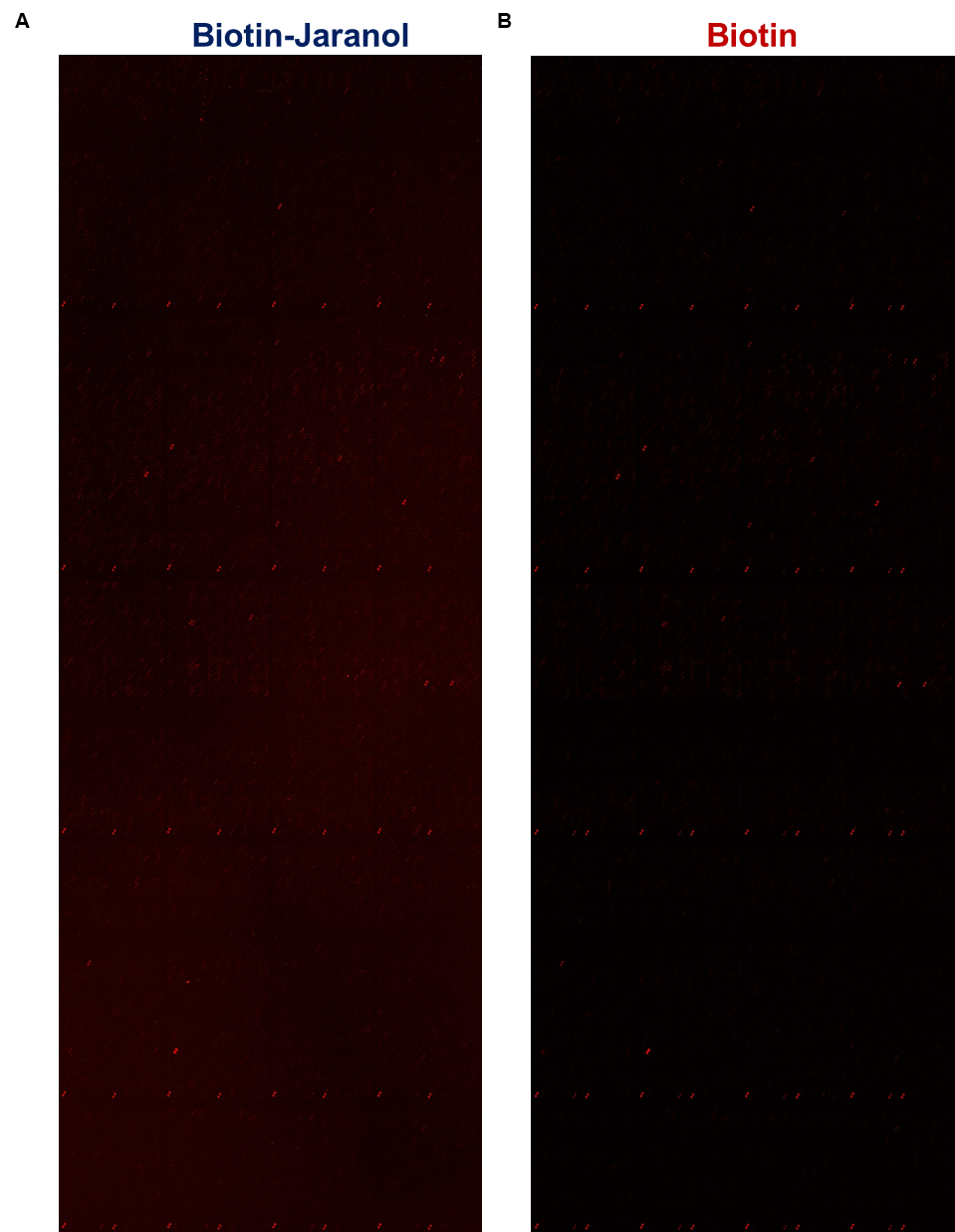


**Figure S14** Representative jaranol proteome microarray images
